# Seed Metabolomic Landscape Reflecting Key Differential Metabolic Profiles Among Different Wheat Cultivars

**DOI:** 10.3390/metabo15090603

**Published:** 2025-09-10

**Authors:** Kgalaletso Othibeng, Lerato Nephali, Fidele Tugizimana

**Affiliations:** Research Centre for Plant Metabolomics, Department of Biochemistry, University of Johannesburg, Auckland Park, Johannesburg 2006, South Africa; 201702253@student.uj.ac.za (K.O.); Lerato.Nephali@omnia.co.za (L.N.)

**Keywords:** food security, GNPS, metabolomics, seed metabolism, seed quality, wheat, UN SDGs, zero hunger

## Abstract

**Background**: Adverse environmental conditions and an ever-increasing world population require devising and designing a roadmap for the next generation of wheat crops for high productivity and resilience to climate change. As such, a fundamental understanding of wheat metabolism and molecular descriptors of wheat seed potentials and quality is a sine qua non step. **Objectives**: In this study we investigated the seed metabolomes of five wheat cultivars to identify differential metabolic profiles and cultivar-related metabolic markers. **Methods**: Liquid chromatography-mass spectrometry (LC-MS) combined with computational strategies and functional analyses was applied. Metabolites were extracted using methanol, and samples were analysed on an LC-MS/MS system. **Results**: The results revealed that the extracted wheat cultivar seed metabolomes spanned a broad range of metabolite classes, including alkaloids, sugars, phenolics, amino acids, hormones, TCA compounds and lipids. Furthermore, the results also revealed key metabolic markers differentiating the wheat cultivars from one another, such as lipids (i.e., MGMG and 13-HODE) and flavonoids (i.e., rutin, tricin and vitexin), amongst many others. **Conclusions**: Such insights are important in assessing seed quality as well as in the selection of markers for seed nutrient and quality trait improvement in wheat breeding programmes. As such, this work generates novel actionable knowledge, a comprehensive metabolomic landscape of wheat seeds and potential markers for cultivar differentiation and quality assessment, which is essential for sustainable and improved wheat production. Thus, the study contributes towards the realisation of sustainable food security, an urgent call for action in a global partnership, as articulated in the United Nations Sustainable Development Goals, particularly zero hunger.

## 1. Introduction

After maize, wheat serves as the most important cultivated staple crop across the globe, and particularly in South Africa, with the common bread wheat (*Triticum aestivum*) accounting for 95% of annual wheat production and durum wheat (*T. turgidum* ssp. durum) making up the remaining wheat cultivation [1,2]. Wheat is a major source of energy and starch, and in addition to this, it also provides substantial amounts of essential vitamins (i.e., B vitamins), protein, phytochemicals, and dietary fibre [3]. In the last decade, there has been a growing effort to improve bread wheat varieties [1,4]. Multidisciplinary research efforts have mainly focused on increasing the quality as well as the yield of wheat and ultimately improving global food security. This focus on higher yields, however, has in turn led to the neglect of other good wheat characteristics such as nutrition and health benefits as well as a loss of biodiversity, which will result in difficulties in meeting future agricultural demands [5,6].

Currently, the challenges of feeding an ever-growing world population, with the food demand expected to rise by 2–5 fold by 2050, and the negative effects of climate change are necessitating the improvement of existing crops as well as the development of new crops of improved yield and nutritional quality and with enhanced resilience to environmental stresses [7,8]. One way to address this would be to improve seed quality, as seeds are the most crucial input for crop production and can influence agricultural productivity [9]. Seed quality embodies different aspects, including seed vigour and germination, genetic purity, and physical, physiological and health quality of plants. High-quality seeds are characterised by (i) complete and rapid germination, (ii) reduced sensitivity to external factors and having the ability to germinate under a broad range of agroclimatic conditions, and (iii) production of normal and vigorous seedlings [10].

Owing to the importance of seed quality in agricultural productivity and human nutrition, there is a growing need in the seed industry for extensive studies to evaluate seed quality and accurately predict seed performance under different conditions [9,10]. Omics sciences such as genomics, transcriptomics, proteomics, and metabolomics can be applied in efforts to investigate and improve seed quality and nutritional traits. Although the abovementioned omics strategies can be applied independently of each other, their integration has been suggested as an effective strategy for the identification of key regulators of various seed quality and nutrient content traits and the genes governing them [11]. Such integration and the insights generated thereof from such studies can contribute significantly to breeding efforts. For example, the identification of potential genes governing seed quality and nutrition traits of interest (i.e., seed weight, colour and size, total amino acid and protein content, disease, and abiotic stress resistance) is of utmost importance in genomic selections for quality trait improvement in breeding programmes. This will in turn lead to the development of high-quality seeds with augmented health benefits as well as the potential of increased and sustainable crop production and ultimately sustainable food security [11,12]. Despite these advances, progress in wheat breeding at the metabolomic level remains limited. Unlike model plants such as Arabidopsis or crops such as rice and maize, the wheat metabolome is less comprehensively characterised due to the complexity of the plant metabolome itself as well as its polyploid genome and strong environmental influences on metabolite accumulation [13,14]. As a result, metabolite annotation in wheat is still incomplete, and the integration of metabolomic data with genomics and phenomics to develop predictive models for breeding has been slow to advance. These knowledge gaps currently limit the translation of metabolomic insights into practical breeding tools.

Metabolomics approaches have been previously applied to investigate the metabolic composition and regulation networks in plants, thus generating new information that could potentially be used to inform crop breeding for improved crop plant traits [9,10,15]. Although current research on the seed metabolome is still limited, some of the studies performed include a comparative metabolomics study by Guo and colleagues on rice seed varieties during germination which revealed that metabolites such as polyamines, flavonoids and amino acids were positively correlated to seed germination [16]. Another study by [17] investigated metabolic changes in seeds and their associations with seed storability in rice. In the case of wheat seeds, an untargeted metabolomics study by Das and colleagues (2017) revealed key metabolite markers in the seed metabolomes of dormant, i.e., pre-harvest sprouting (PHS)-tolerant, and non-dormant (PHS-susceptible) wheat cultivars following water imbibition. The results revealed differential expression of lipids, plant hormones, amino acids, and oxalate in response to imbibition of both dormant and non-dormant wheat seeds [18].

Overall, plant metabolomics studies on seeds of major crops such as maize, tomato and rice have shed remarkable insights into the metabolome composition, significant metabolite-metabolite correlations, metabolite abundance variations as well as regulation of seed metabolism in these crop plants [19,20,21]. Thus, herein we report on a liquid chromatography-mass spectrometry (LC-MS)-based untargeted metabolomics work, involving computational omics strategies, to decode the metabolomic landscape of five wheat seed cultivars and investigate the differential metabolic profiles among these cultivars. Specifically, this study aimed to (i) identify metabolite markers that differentiate wheat cultivars with respect to seed quality traits, (ii) evaluate their potential relevance to agronomically important characteristics such as germination vigour and nutritional content, and (iii) provide a metabolomic resource that can be integrated into marker-assisted breeding and trait prediction models. By linking metabolic profiles to seed quality attributes, this work paves a necessary foundation for the development of high-quality wheat seeds with improved resilience and nutritional value, thereby supporting sustainable wheat production and ultimately sustainable food security.

## 2. Materials and Methods

Seeds of five (5) common wheat (*Triticum aestivum*) cultivars (or varieties), namely, ‘Koonap’, ‘Gariep’, ‘Matlabas’, ‘Senqu’ and ‘Elands’, were obtained from the Small Grain Institute of the Agricultural Research Council (ARC-SGI) in Bethlehem, Free State Province, South Africa. The wheat varieties under study have different release dates; for example, Gariep was released in 1993, Elands in 1998 and Matlabas in 2004, while Koonap and Senqu were both released in 2012. The cultivars under study can also be categorised based on their growth type; for example, Matlabas fall under winter-type wheats, while Elands, Senqu, Gariep and Koonap are intermediate-type wheats (https://www.arc.agric.za/arc-sgi, accessed on 4 September 2022). Moreover, they also differ in their cultivar characteristics (Table 1), such as yield potential and stability, and their adaptability to the area of production, as well as in agronomic traits, such as resistance/susceptibility to aluminium toxicity, pests and diseases [22].

### 2.1. Metabolite Extraction and Sample Preparation

Mature seeds of the 5 cultivars were crushed (using a blender) to powder form and stored until metabolite extraction. To extract metabolites, 2 g of the crushed seeds were weighed and dissolved in 10 mL of cold 80% analytical grade methanol (1:10 *m*/*v*) prior to being subjected to sonication for 60 s at 55% power using a probe sonicator (Bandelin Sonopuls, Berlin, Germany). The use of methanol as an extraction solvent is well-established in plant metabolomics studies, as it helps to extract a broad range of metabolites, including polar and mid-polar metabolites [23]. The methanol extracts were then centrifuged at 4000 rpm for 20 min, following which the supernatants were concentrated to approximately 1 mL using a Büchi Rotavapor R-200 (Heidolph Laborota, Schwabach, Germany) at 55 °C and then dried to completeness overnight at 45 °C using a speed vacuum concentrator (Eppendorf, Merck, Johannesburg, South Africa). The dried residues were resuspended in 500 µL of LC-MS-grade methanol: milliQ water (1:1, *v*/*v*) and then filtered into HPLC vials (Shimadzu, Johannesburg, South Africa) and stored at 4 °C until further analysis. For each of the cultivars, 3 biological replicates were prepared, and quality controls comprising pooled equal volumes of all seed cultivar samples were also prepared.

### 2.2. Liquid Chromatography-Quadrupole Time-of-Flight Tandem Mass Spectrometry

The method followed for sample analysis (data acquisition) was exactly as described in [24]. Briefly, the aqueous-methanol extracts were analysed using a liquid chromatography-quadrupole time-of-flight tandem mass spectrometry instrument (LCMS-9030 qTOF, Shimadzu Corporation, Kyoto, Japan) for the untargeted approach. The samples were first separated chromatographically on a Shim-pack Sceptor C18 column (2.1 × 100 mm with a particle size of 1.9 µm) (Shimadzu Corporation, Kyoto, Japan) at 55 °C. A sample volume of 3 µL was injected, and a binary solvent gradient system was used consisting of solvents A: 0.1% formic acid in milliQ water (both HPLC grade, Merck, Darmstadt, Germany) and B: (0.1% formic acid in acetonitrile (HPLC grade, Romil SpS, Cambridge, UK). A flow rate of 0.3 mL/min was used throughout the 53 min total chromatographic run time, and the separation was performed as follows: the initial conditions (10% solvent B) were maintained for 3 min, followed by a gradual change from 10 to 60% solvent B over 3–40 min; the conditions were maintained at 60% solvent B for 3 min (from 40 to 43 min) and then changed to 90% solvent B between 43 and 45 min, which was maintained for 3 min. The gradient was then returned to initial conditions between 48 and 50 min, following which the analytical column was allowed to calibrate for 3 min prior to the next injection. Each sample was analysed in triplicate.

Further analysis of the chromatographic effluents was performed using the qTOF high-definition mass spectrometer with electrospray ionisation (ESI) operating in the negative mode. Based on preliminary optimisation, the ESI negative gave better ionisation efficiency and coverage and was therefore the ionisation mode of choice for this study. The subsequent MS parameters used were as follows: interface temperature of 300 °C and interface voltage of 4.0 kV, heat block temperature of 400 °C, nebulisation and dry gas flow of 3 L/min, DL temperature of 280 °C, flight tube temperature of 42 °C and a detector voltage of 1.8 kV. To monitor high mass accuracy, the calibration solution, sodium iodide (NaI), was used. Data-dependent acquisition (DDA) was performed where both MS1 and MS2 (unfragmented and fragmented, respectively) data were simultaneously generated for all ions falling within an *m*/*z* range of 100–1000 and with an intensity threshold greater than 5000 counts. For the fragmentation experiments, the collision gas used was argon at a collision energy of 30 eV with a 5 eV spread. The prepared QC samples were used to condition the LC-MS system as well as for non-linear signal correction. Moreover, to ensure system equilibration, the QC samples were injected at the beginning and end of the batch. Sample acquisition was randomised, and the QC sample was analysed every 10 injections to monitor and correct changes in the instrument response.

For targeted analysis, a triple quadrupole mass spectrometry platform, LCMS-8050 (Shimadzu, Kyoto, Japan), equipped with an ESI source and ultra-fast liquid chromatography (UFLC) as a front-end, was used. The different chromatographic separation conditions for the targeted amino acids, flavonoids, hormones and other phenolics are summarised in Tables S1–S4. Moreover, absolute quantification of the targeted metabolites was performed using a multiple reaction monitoring (MRM) method with the conditions provided in Table S5.

### 2.3. Data Mining: Data Pre-Processing and Chemometrics Analyses

For the untargeted LC-MS analysis, the acquired raw data were processed using the web-based processing platform, XCMS Online (version 3.7.1) [25], with the following parameters: feature detection was performed using the CentWave method, the maximal tolerated *m*/*z* deviation was set to 30 ppm, the minimum and maximum peak widths were set to 10 and 60, respectively, the signal/noise threshold was set to 6 and the noise filter and prefilter intensity were set to 0 and 100, respectively. For the retention time correction, the obiwarp method was used with a profStep of 1, and for alignment, the bandwidth was set to 5, the minfrag (i.e., the minimum fraction of sample necessary in a group) was 0.5 and mzwid (*m*/*z* width for determining peak groupings). Moreover, a statistical test was performed using the Kruskal–Wallis non-parametric method, and post hoc analysis was also performed, with the ***p***-value threshold set to 0.01. The resultant feature table with 12,991 features was imported into the SIMCA (soft independent modelling of class analogy) software, version 17.0 (Sartorius, Johannesburg, South Africa). Data scaling was performed using Pareto scaling, which makes use of the square root of the standard deviation as the scaling factor [26]. Unsupervised models such as principal component analysis (PCA) and the hierarchical clustering algorithm (HCA) were explored and presented in this study, highlighting patterns within the datasets. Then, based on the insights extracted from unsupervised modelling, partial least squares-discriminant analysis (PLS-DA), a supervised method, was applied to the Pareto-scaled data, allowing for sample classification as well as the identification and selection of variables underlying the discrimination between wheat cultivar seeds, using the MetaboAnalyst 5.0 Bioinformatics Suite [27]. In addition to the multivariate statistical modelling, univariate statistical tests such as *t*-tests were also applied where necessary, and this is indicated in the results section.

In the case of targeted analysis, processing of the raw centroid data was performed as follows: The UHPLC-qTOF-MS raw data were processed using the MarkerLynx application of MassLynx XS™ software, version 4.1 (Waters, Manchester, UK), which makes use of the patented ApexTrack algorithm [28] to perform accurate peak detection and alignment and results in a data matrix of retention time (Rt) *m*/*z* variable pairs, with *m*/*z* peak intensity for each sample. The following parameters were used for data processing: a retention time (Rt) range of 1–17 min, a 100–1100 Da mass range, an intensity threshold of 50, a mass tolerance of 0.05 Da, and an Rt window of 0.2 min for both polarities. Normalisation was then performed by using total ion intensities of each defined peak; prior to calculating intensities, the software performs patented modified Savitzky–Golay smoothing and integration. Only data matrices with noise levels below 50% (MarkerLynx metrics) were used for downstream data analysis strategies. The data matrices generated from MassLynx were exported into the MetaboAnalyst (version 5.0) [27] bioinformatics suite for relative quantification (heatmap analysis). The generated matrices (from both targeted and untargeted analyses) were then exported into the MetaboAnalyst web-based Bioinformatics Suite to generate relative quantification heatmaps, prior to which data normalisation was performed by Pareto scaling and log transformation.

### 2.4. Metabolite Annotation and Biological Interpretation

The raw vendor (i.e., Waters) format MS/MS data were first converted to ‘analysis base file’ (ABF) format using the Reifys Abf converter software (https://www.reifycs.com/AbfConverter/, accessed on 20 May 2022) and then uploaded into the Mass Spectrometry-Data Independent Analysis (MS-DIAL) software, version 4.9. Data processing with MS-DIAL was carried out as detailed in [29], following which the resultant GNPS export files, i.e., GnpsMgf and GnpsTable (feature quantification table), were then uploaded into the GNPS environment (https://gnps.ucsd.edu/, accessed on 20 May 2022) using the WinSCP server for molecular networking.

Classical and feature-based molecular networks (FBMN) were generated for both the ESI-negative and positive mode data of the roots and leaves by uploading the respective MGF file, and in the case of FBMN, in addition to the MGF file, a feature quantification table and a metadata file describing the different cultivars under study were also uploaded. It is worth noting that a classical MN job was performed for comparing ion distribution across different wheat cultivars and that, instead of a metadata file, the MGF files of the different wheat cultivars were uploaded under different groupings (i.e., G1–G2). For classical MN, the MS/MS (fragmentation) spectra were clustered using the MS-Cluster algorithm with a precursor ion mass tolerance of 0.1 Da and a fragment ion mass tolerance of 0.1 Da to create the consensus spectra, and precursor ion and fragment ion mass tolerances of 0.05 Da were used for FBMN. For both FBMN and classical MN (ESI positive and negative), a network was generated where the edges connecting the nodes were filtered to have a cosine score above 0.7 and a minimum of four corresponding fragment ions. The MN spectra were also searched against the spectral libraries housed in GNPS, where the same parameters (i.e., cosine score > 0.7 and min-matched fragments of 4) were used for metabolite annotation.

Moreover, MS2LDA jobs were performed to gain insight on the substructural diversity in the extracted wheat seed metabolomes. The parameters used for the MS2LDA_MOTIFDB workflows were as follows: bin width was set to 0.005, the number of LDA iterations at 1000, the minimum MS2 intensity at 100 and LDA free motifs at 300, and GNPS, Massbank, Euphorbia and Urine MotifDB motif databases within the GNPS platform were included. Additionally, in silico annotation tools, NAP and DEREPLICATOR+, were also used to improve upon the annotation and in turn enhance the chemical insight gained from the extracted lead and root metabolomes. Structure databases used for NAP included GNPS, SUPNAT, DNP, HMDB and CHEBI, as well as a user-provided database, while DEREPLICATOR+ jobs were performed using the respective MGF files and parent as well as fragment ion tolerances of 0.005 Da.

The resultant molecular network data were then enhanced with the MolNetEnhancer workflow, which combines the output from FBMN, MS2LDA, NAP and DEREPLICATOR+ to improve the chemical structural annotations acquired before they were visualised using the Cytoscape network visualisation software (version 3.8.2), where the nodes and edges were labelled and coloured. Classical MN networks composed of nodes labelled with the precursor mass (passthrough mapping) which were also coloured based on the different wheat seed cultivars (Senqu, Matlabas, Gariep, Elands and Koonap). For the FBMNs, the nodes were labelled with the precursor mass (*m*/*z*) and coloured by means of pie charts based on the differential changes in the metabolite levels under different wheat cultivar seed metabolomes. For the MolNetEnhancer networks, the nodes were also labelled with the precursor mass (*m*/*z*) but coloured based on the superclasses such that nodes present in the same superclass had the same colour, while grey nodes represented the non-matched metabolites. The fragmentation spectra of all the putatively annotated metabolites matched to the GNPS spectral libraries were manually validated using the metabolite annotation workflow described in [29].

In the current study, metabolites were putatively annotated to level 2 of the Metabolomics Standards Initiative (MSI) [30]. All untargeted and targeted metabolites (Table S6) were used for metabolic pathway analysis. Metabolic pathway analysis was performed with the Metabolomics Pathway Analysis (MetPA) component of the MetaboAnalyst bioinformatics tool suite (version 5.0). This enabled the identification of the affected metabolic pathways, analysis thereof, and visualisation. MetPA uses high-quality KEGG metabolic pathways as the backend knowledge base. In addition to the existing literature, the use of these bioinformatics tools (for pathway analysis) provided a framework to partially map the molecular landscape of the metabolism under study, enabling the biological interpretability of observed changes in a metabolome view [31]. Furthermore, correlation network analysis was also performed using MetaMapp (version 2.0.1.45) [32]. This was performed using MetaMapp-encoded chemical structures of the annotated metabolites obtained from KEGG and PubChem databases, as well as fold changes and *p*-values obtained from descriptive statistics (Table S7). Similarity cut-off among metabolites was defined using a Tanimoto score threshold of 0.7, and the generated metabolic networks were visualised using the Cytoscape network visualisation software (version 3.8.2) [32].

## 3. Results

As detailed in the experimental section (Section 2), the metabolomes of dry seeds belonging to five (5) wheat cultivars namely, Koonap, Gariep, Matlabas, Senqu and Elands, were profiled, using an untargeted approach, with an LC-MS system as the analytical platform. Such metabolic profiles provide fundamental and actionable insights for seed management and quality control. Moreover, as highlighted in Section 2, the wheat cultivars under study differ in their growth type, that is, ‘Koonap’, ‘Gariep’, ‘Senqu’ and ‘Elands’ are intermediate-type cultivars, while ‘Matlabas’ falls under winter-type wheats. The results are presented under three subsections: Section 3.1 provides a chemometric overview of the wheat cultivar seed, highlighting sample groupings and patterns observed, followed by annotation of the wheat seed metabolomes, while Section 3.2 focuses on relative quantification and differentiation of the metabolome of the wheat cultivar seeds. In the last section, Section 3.3, the annotated metabolites are mapped into biological pathways to describe the metabolic potentials of the seeds. Furthermore, the correlation networks are generated to decipher the metabolic relationship patterns.

### 3.1. Chemometric Analyses and Metabolic Profiling of Wheat Cultivar Seed Metabolomes

**Figure 1:**
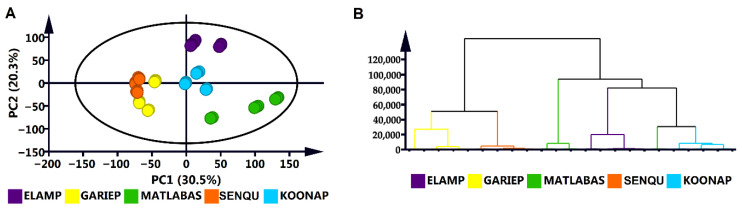
Unsupervised modelling: (**A**) A PCA score plot of a 7-component model explaining 84.3% of the total variation in Pareto-scaled, ESI-negative data with 77.8% predictive power (Q^2^), showing wheat cultivar-related grouping. (**B**) A hierarchical clustering analysis (HCA) dendrogram showing cultivar-related sample clustering, corresponding to the PCA scores plot in (**A**).

To describe key metabolic markers and profiles that differentiate the five wheat cultivars, chemometric methods were applied. Firstly, unsupervised methods, namely principal component analysis (PCA) and hierarchical clustering analysis (HCA), were used to reduce (metabolomics) data dimensionality and to identify patterns and inherent structures within the data. The PCA scores plot (Figure 1A) revealed cultivar-related grouping, pointing to differences and similarities between and within the different cultivars. This was complemented by the HCA dendrogram (Figure 1B), which mimicked the observations made from the PCA scores plot and, in addition, revealed how the cultivar groupings are chemically related to or separate from one another.

**Figure 2:**
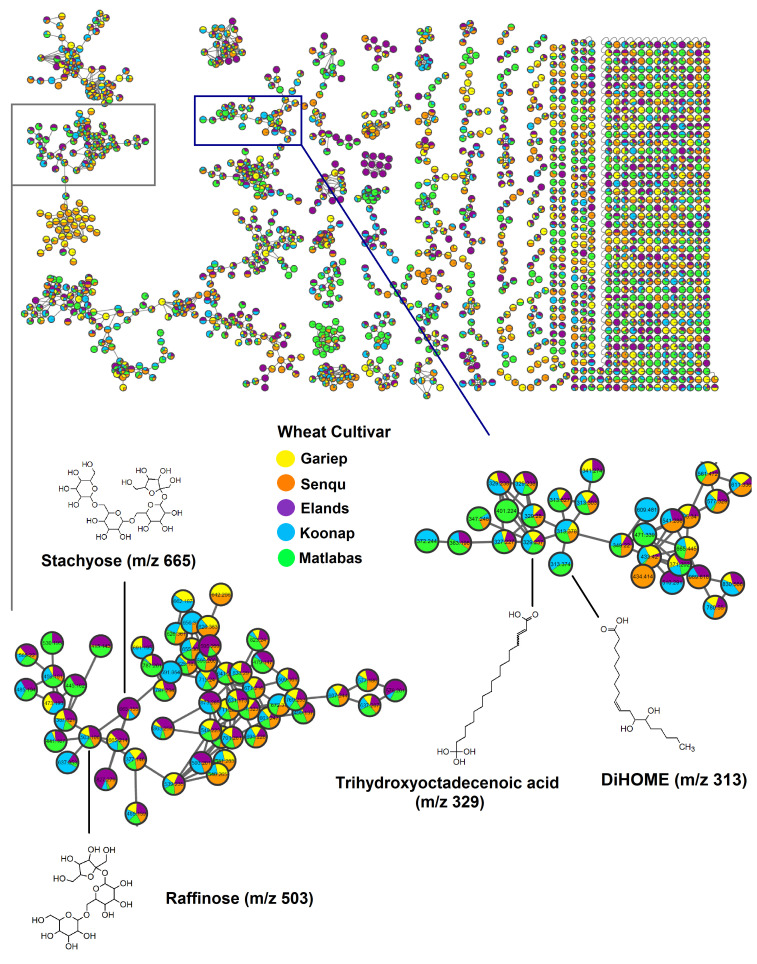
Classical molecular network of wheat seed extracts. MN constructed from ESI negative spectral data, shows both the full network and extracted clusters. The zoomed-in lipids and sugars subnetworks display (selected) chemical structures of GNPS library-matched metabolites. The node colours represent the different wheat cultivars under study, namely, the Elands, Gariep, Koonap, Matlabas and Senqu cultivars.

Moreover, to comprehensively investigate the seed metabolome of the different wheat cultivars, molecular networking (MN) approaches were applied, providing a metabolic overview of the extracted metabolomes. Molecular networking follows the principle that structurally similar metabolites give rise to similar fragmentation spectra and makes use of various algorithms, such as the MS-cluster algorithm, to organise experimental spectra (represented by nodes) into molecular clusters (or molecular families) based on spectral similarities [33]. The classical MN approach, which relies solely on MS2 information for clustering, was first applied, and the computed network had a total of 1721 nodes of consensus spectra, with 1120 nodes clustered into 33 independent molecular families of structurally similar metabolites formed by two or more nodes connected by edges (Figure 2). Moreover, consensus spectra which were not clustered into molecular families were represented as singletons (i.e., self-loop nodes) at the bottom of the network as illustrated in Figure 2. From the classical MN network, 115 nodes were putatively annotated through spectral matching to libraries housed within the GNPS ecosystem. As mentioned in the experimental section (Section 2), for a query spectrum to be matched to library spectra, parameters such as the minimum number of matched peaks (i.e., the number of common fragment ions between the experimental and library spectra) and the cosine score threshold are taken into consideration.

In the classical MN output visualised using Cytoscape, the nodes were coloured based on the different wheat cultivars, and the pie charts represent the MS2 spectral counts indicative of the presence or absence of the metabolites in the respective cultivars. As observed in the classical MN results (Figure 2), the presence of metabolites (nodes) in wheat cultivar seeds varied across the different clusters (molecular families), with some clusters comprising metabolites present in all cultivars and others comprising metabolites only present in some cultivars but absent in others. For example, in the zoomed-in lipids subnetwork, trihydroxyoctadecenoic acid (*m*/*z* 329.237) was present in all five cultivars (i.e., Senqu, Matlabas, Koonap, Gariep and Elands), while DiHOME (*m*/*z* 313.374) was found to be present in Matlabas and Koonap cultivars only. Moreover, in the sugars cluster, raffinose (*m*/*z* 503.106) was also present in all five cultivars, while stachyose (*m*/*z* 665.155) was present in all cultivars except Matlabas and had the highest relative spectral count in the Elands cultivar in comparison to other cultivars.

**Figure 3:**
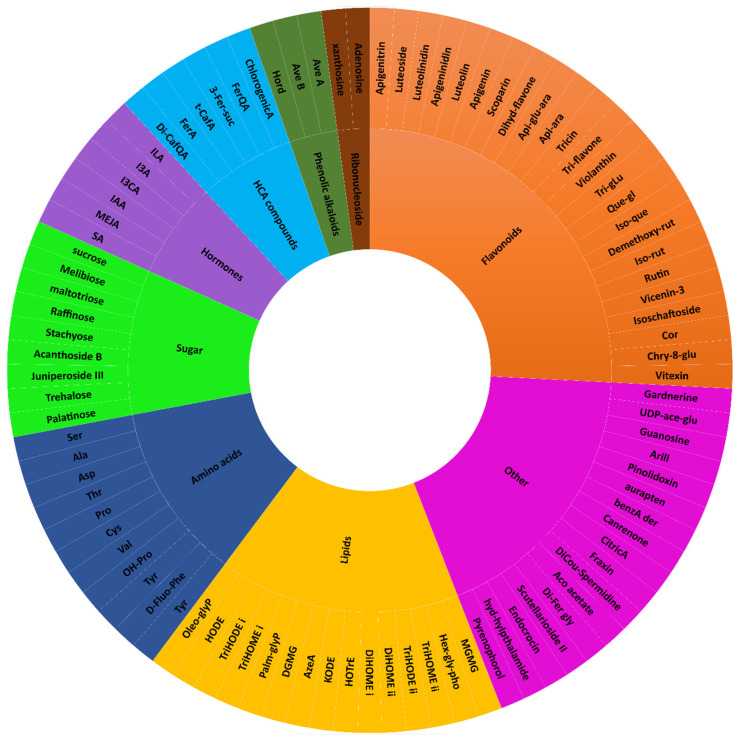
Metabolome coverage of wheat seed cultivars. A sunburst plot displaying all targeted and putatively annotated metabolites from dry seeds of Elands, Gariep, Koonap, Matlabas and Senqu wheat cultivars. Full metabolite names are provided in Table S8.

The MolNetEnhancer results revealed that the extracted wheat cultivar metabolomes spanned a wide range of chemical superclasses denoted by coloured nodes, while the unknown superclasses with no matches were denoted by grey nodes (Figure S1). The putatively annotated superclasses across the five cultivars included benzenoids, organic acids and derivatives, alkaloids and derivatives, phenylpropanoids and polyketides, lipids and lipid-like molecules, organic nitrogen compounds, organic polymers, nucleosides, nucleotides and analogues, lignans, neolignans and related compounds, organic oxygen compounds, organohalogen compounds and organoheterocyclic compounds (Figure S1). Thus, harnessing the power of molecular networking strategies, in addition to selected amino acids, hormones, flavonoids and alkaloids that were quantified in targeted analyses (Section 2), we were able to annotate a total of 41, 42, 40, 47 and 47 metabolites from the Elands, Gariep, Koonap, Matlabas and Senqu cultivars, respectively (Table S6). These metabolites span various chemical classes of both primary and secondary (specialised) metabolism, with differential profiles across the five wheat cultivars (Figure 3 and Figure S1).

Out of the 93 annotated metabolites (across the seeds of five wheat cultivars), 38 metabolites were of primary metabolism (i.e., amino acids, sugars, tricarboxylic acid (TCA) compounds, nucleic acid analogues, fatty alcohol, and lipids), while the majority, 55 compounds, are secondary (specialised) metabolites (i.e., alkaloids, hydroxycinnamic acid (HCAs) compounds, flavonoids, hormones, steroids and other phenolic compounds) (Figure 3). The presence and absence of the putatively annotated metabolites (i.e., belonging to different classes) differed across the five wheat cultivar seeds studied. For example, in the case of lipids, the Matlabas seed metabolome had the highest number of lipids (15), followed by Koonap with 12 lipids, Senqu with 11 and Gariep with 10, while Elands seeds were found to have the least number of lipids annotated (8). Interestingly with regard to flavonoids, Senqu seeds were found to have the highest number of (putatively annotated) flavonoids (20), while Koonap and Matlabas seeds were both found to have the least flavonoids (14), and Elands and Gariep seeds had 16 and 17 annotated lipids, respectively. Additionally, wheat seeds belonging to Koonap, Matlabas and Senqu cultivars were found to have a total of five putatively annotated hydroxycinnamic acid (HCAs) compounds and derivatives, while Elands seeds had a total of eight putatively annotated HCAs compounds and derivatives, followed by Gariep seeds with six. In the case of sugars, Koonap seeds had the highest number of sugar annotations with nine sugars, followed by Gariep, Senqu and Matlabas seeds with seven and Elands seeds with five putatively annotated sugars. Moreover, seeds belonging to Elands, Koonap and Senqu wheat cultivars were found to have a total of 11 amino acids each, while Gariep and Matlabas seeds had 10 amino acids each.

### 3.2. Differentiation of the Different Wheat Cultivar Seeds

**Figure 4:**
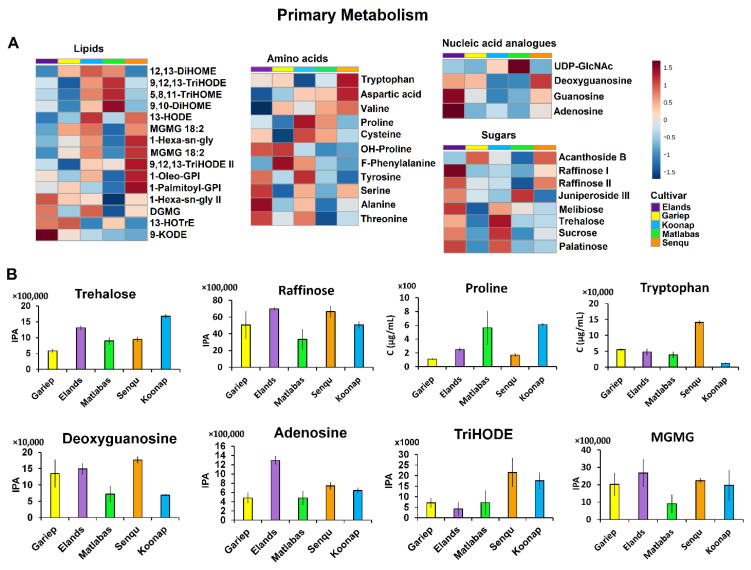
Differential quantitative changes in levels of primary metabolites. (**A**) Heatmaps and (**B**) bar graphs, both displaying differential quantitative alterations in the levels of amino acids, lipids, sugars, and nucleic acid analogues across the different wheat cultivar seeds under study (i.e., Senqu, Matlabas, Elands, Koonap and Gariep). Full metabolite names are provided in Table S6.

**Figure 5:**
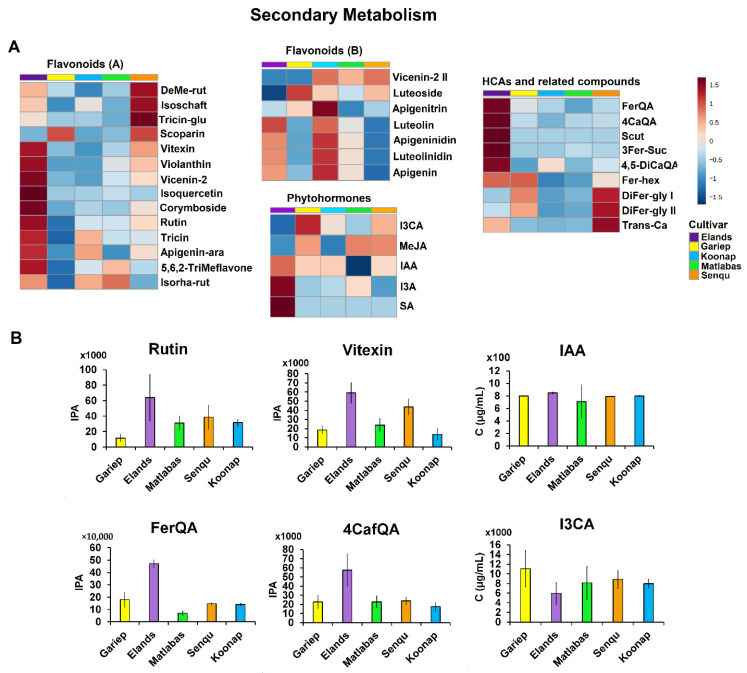
Relative quantification of altered specialised (secondary) metabolites. (**A**) Heatmaps and (**B**) bar graphs both displaying differential changes in quantitative levels of secondary metabolites such as phytohormones, flavonoids and hydroxycinnamic acid (HCAs) compounds and related compounds across different wheat cultivar seeds. Full metabolite names are provided in Table S6.

Identifying and quantifying changes in the extracted wheat seed metabolomes can reveal significant metabolite variations across the different wheat cultivars and provide valuable insights regarding the seed metabolism, which may aid in understanding and improving seed quality and seed potentials [34]. Thus, heatmaps of all the annotated metabolites (i.e., from both targeted and untargeted analyses) were generated to further explore the observed similarities and differences in the wheat cultivar metabolite profiles and for relative quantification. The differential quantitative changes spanned both the primary and secondary plant metabolism (Figure 4 and Figure 5).

#### 3.2.1. Primary Metabolism in Dry Wheat Seeds

The levels of primary metabolites such as amino acids varied across the five (5) cultivar seeds under study (i.e., Gariep, Elands, Koonap, Matlabas and Senqu cultivars), as shown in Figure 4A,B. Notably, the levels of proline were higher in the Matlabas and Koonap seeds and lowest in Gariep cultivar seeds (*p*-value Gariep vs. Koonap = 1.49 × 10^−12^; Gariep vs. Matlabas = 2.04 × 10^−4^), while tryptophan levels were the highest in Senqu seeds and lowest in Koonap seeds (*p*-value Senqu vs. Koonap = 4.51 × 10^−12^; Figure 4B). Sugar levels were higher in Elands seeds, followed by Koonap seeds, compared to Gariep, Senqu and Matlabas seeds (Figure 4A). Moreover, in the case of trehalose, Koonap seeds had the highest levels, while Gariep seeds had the lowest levels of the sugar (*p*-value Gariep vs. Koonap = 2.81 × 10^−17^; Figure 4B).

Additionally, lipids such as MGMG, HODE, TriHODE and 1 Oleo GP I were found in higher levels in Senqu, whereas 9,10-DiHOME, 9,12,13-TriHODE and TriHOME were found in higher levels in Matlabas compared to other cultivars (Figure 4A). Moreover, as shown in Figure 4B, Senqu seeds were found to have the highest levels of TriHODE, while Elands seeds had the lowest levels (*p*-value Senqu vs. Elands = 1.83 × 10^−4^). MGMG levels, on the other hand, were the highest in Elands seeds and the lowest in Matlabas seeds (*p*-value Matlabas vs. Elands = 4.70 × 10^−5^). Furthermore, the levels of nucleic acid analogues were relatively higher in Gariep, Elands and Senqu cultivars and lower in Matlabas and Koonap seeds (Figure 4A). Notably in Figure 4B, the levels of deoxyguanosine were found to be the highest in Senqu seeds, followed by Elands seeds, and the lowest in Koonap seeds (*p*-value Senqu vs. Koonap = 1.09 × 10^−9^). Additionally, Elands seeds had the highest levels of adenosine, while Gariep and Matlabas seeds had the lowest levels (*p*-value Matlabas vs. Elands = 2.38 × 10^−8^; *p*-value Gariep vs. Elands = 2.90 × 10^−10^).

#### 3.2.2. Specialised Metabolism in Dry Wheat Seeds

The heatmaps of all annotated metabolites also revealed trends in metabolite levels differentiating the wheat cultivars under study from one another. For example, specialised (secondary) metabolites primarily involved in plant defence and signalling, flavonoids and hydroxycinnamic acid (HCAs) had relatively higher levels in Senqu, Elands and Koonap seeds as compared to Matlabas and Gariep seeds (Figure 5A). To be specific, the levels of HCA compounds such as Scut and 4,5-caffeoyl quinic acid were higher in Elands, and two isomers of diferuloyl glycoside (DiFer-gly I and II) and transCa were higher in Senqu (Figure 5A). Moreover, as shown in Figure 5B, Elands seeds had the highest levels of feruloyl quinic acid (FerQA), while Matlabas seeds had the lowest levels of this HCA compound (*p*-value Elands vs. Matlabas = 3.51 × 10^−13^). Similarly, Elands seeds were also shown to have the highest levels of 4-caffeoylquinic acid (4CafQA), which were also found to be the lowest in Koonap seeds (*p*-value Elands vs. Koonap = 1.74 × 10^−4^).

In the case of flavonoids, the levels of glycosylated forms such as vicenin, isoquercetin, apigenin arabinoside and corymboside were relatively higher in Elands compared to the other four cultivars, whereas the antifungal 3-deoxyanthocynidin phytoalexins, apigeninidin and luteolinidin, were higher in Koonap compared to the other cultivars (Figure 5A). These results indicate that Koonap seeds might be more resistant to fungal infection compared to the other cultivars. Additionally, as depicted in Figure 5B, Elands seeds had the highest levels of rutin, while Gariep seeds were found to have the lowest rutin levels (*p*-value Gariep vs. Elands = 3.3 × 10^−4^). Similarly, vitexin levels were the highest in Elands wheat seeds and the lowest in Koonap seeds (*p*-value Koonap vs. Elands = 1.14 × 10^−7^). Additionally, the levels of phytohormones such as indole-3-carboxyaldehyde (I3A) and salicylic acid were relatively higher in seeds belonging to the Elands cultivar as compared to the other four wheat cultivar seeds (Figure 5A). Moreover, as shown in Figure 5B, the levels of indole acetic acid (IAA) were comparably similar across the Gariep, Senqu and Koonap cultivars under study (i.e., *p*-value Senqu vs. Koonap = 0.11), while Elands seeds had comparatively higher levels of IAA than Matlabas seeds (*p*-value Elands vs. Matlabas = 0.07). Interestingly, in the case of indole-3-carboxylic acid (I3CA), Gariep seeds were found to have the highest level of this phytohormone, while Elands cultivar seeds had the lowest levels (*p*-value Elands vs. Gariep = 4.39 × 10^−4^; Figure 5B).

### 3.3. Metabolic Pathway and Network Analyses of the Annotated Wheat Cultivar Seed Metabolomes

**Figure 6:**
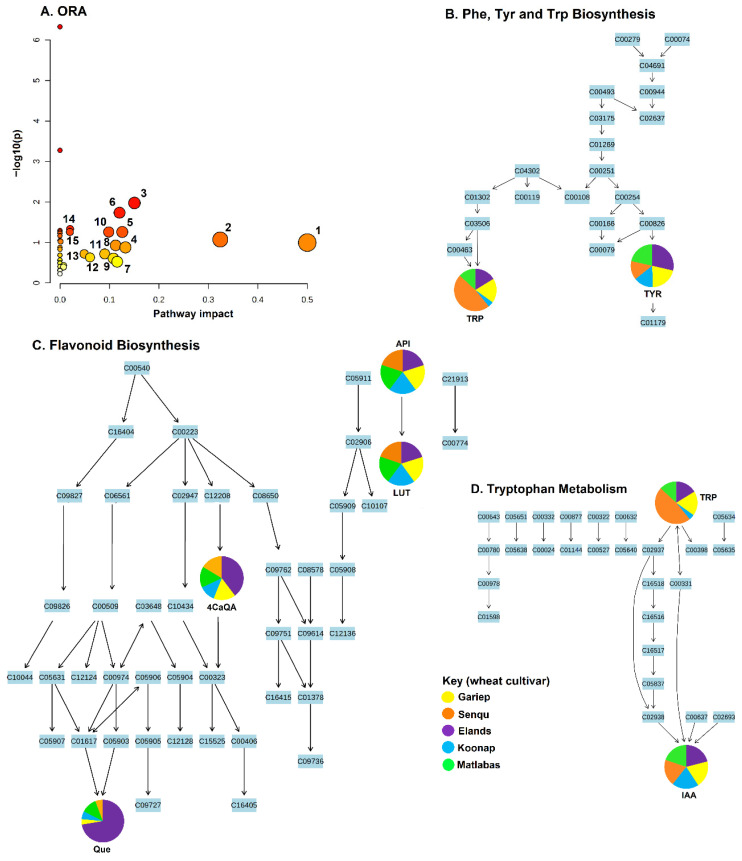
Metabolic pathway analyses. (**A**) The graph displaying the ‘metabolome view’ containing the mapped pathways arranged by pathway impact (differential significance) on the *x*-axis and *p*-values on the *y*-axis. The nodes (circles) represent the mapped metabolic pathways, and the node sizes are reflective of the impact of each pathway. Topological characteristics of some of the significantly impacted pathways are shown, namely the (**B**) phenylalanine, tyrosine, and tryptophan biosynthesis, (**C**) flavonoid biosynthesis, and (**D**) tryptophan biosynthesis pathways. The pie charts indicate differential levels of the mapped metabolites in the different wheat cultivar seeds. (1) isoquinoline alkaloid biosynthesis, (2) tryptophan metabolism, (3) galactose metabolism, (4) stilbenoid, diarylheptanoid and gingerol biosynthesis, (5) alanine, aspartic acid and glutamate metabolism, (6) glycine, serine and threonine metabolism, (7) TCA cycle, (8) arginine and proline metabolism, (9) tyrosine metabolism, (10) starch and sucrose metabolism, (11) phenylpropanoid biosynthesis, (12) sulphur metabolism, (13) cysteine and methionine metabolism, (14) flavonoid biosynthesis, (15) phenylalanine, tyrosine and tryptophan biosynthesis. Abbreviations: Tyr = tyrosine, Trp = tryptophan, Api = apigenin, Lut = luteolin, IAA = 3-indoleacetic acid, Que = quercetin, 4CaQA = 4-Caffeoylquinic acid.

Biologically, the metabolites in the extracted seed metabolomes are involved in several important biological pathways, as revealed by a functional analysis, infographically depicted in Figure 6. Metabolic pathway analysis was performed using the over-representation analysis (ORA) method in the MetaboAnalyst 5.0 bioinformatics suite. The obtained results revealed isoquinoline alkaloid biosynthesis, tryptophan metabolism, galactose metabolism, flavonoid biosynthesis, the TCA cycle, stilbenoid, diarylheptanoid and gingerol biosynthesis, and alanine, aspartic acid and glutamate metabolism as the most significantly impacted pathways, amongst others (Figure 6).

**Figure 7:**
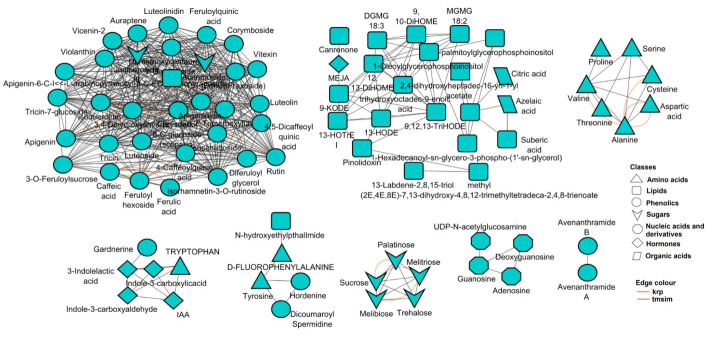
A correlation-based metabolic network. The network reveals complex metabolic relationship patterns between all the annotated metabolites in the Gariep vs. Senqu wheat cultivar seeds. Each metabolite is represented by a node, and the metabolites are connected based on structural similarity (tmsim, Tanimoto similarity) and biochemical relationships (krp, KEGG reactant pairs). The node shapes represent the classes the metabolites belong to.

Furthermore, considering that metabolic pathways are functionally interconnected, correlation metabolic network analysis was also performed, which revealed complex relationships (i.e., interconnectedness) between the measured metabolites (Figure 7). In the computed network, each node represents a metabolite, and the edges connecting the different nodes correspond to the mathematical correlation levels between the metabolite pairs [35,36]. Moreover, the different node shapes correspond to the metabolic classes the annotated metabolites belong to (Figure 7).

**Figure 8:**
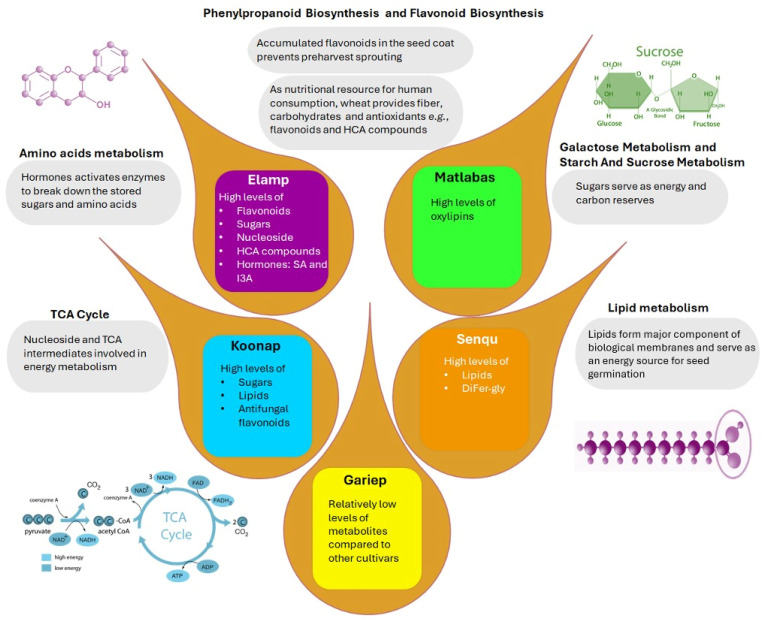
An overview of the metabolomic landscape of wheat seeds. Different cultivars have different metabolite profiles. The differential metabolites are functionally involved in different biological pathways. These differences would define the cultivar-related nuances in seed quality, growth and germination.

Furthermore, this functional analysis (Figure 7) reveals high interconnectedness of all the annotated metabolites, independent of the individual biological pathways (indicated by pathway analysis, Figure 6). The metabolites were interconnected based on their chemical relationships, as indicated by the grey edges, and by KEGG reactant pairs, as shown in orange. Such interconnectivity demonstrates correlated biochemical and structural metabolic relationships that coordinate the wheat seed metabolism. Topologically, eight distinct hubs of diverse metabolite classes, such as amino acids, hormones, lipids, sugars, nucleic acids and phenolics, are observed, with phenolics forming the biggest cluster, followed by the lipids cluster (Figure 7). Thus, the methodology applied in this study evidently captured comprehensively (for the first time) the metabolomic landscape of wheat seed as well as essential components that define the seed metabolism of the five wheat cultivars. Such insights suggest also that the five wheat cultivars are metabolically different and distinguishable (Figure 8).

## 4. Discussion

In this study, an untargeted metabolomics approach was applied to profile the metabolomes of dry seeds of Elands, Matlabas, Koonap, Senqu and Gariep wheat cultivars. Both the PCA scores plot and HCA dendrogram (Figure 1) revealed clustering of the Gariep and Senqu cultivars closer to one another, indicating similarities in the underlying metabolite profiles as compared to the other three wheat cultivars (i.e., Koonap, Elands and Matlabas). According to the wheat breeding guidelines provided by the ARC-LNR (https://www.arc.agric.za/arc-sgi, accessed on 4 September 2022), although all five cultivars are dryland wheat cultivars and are all sourced from summer rainfall regions, they do, however, differ in their growth type. It can be postulated that such differences in the physiology and growth behaviour of these cultivars reflect nuances in their metabolism, which correlate to differential metabolic profiles suggested by the PCA and HCA models (Figure 1).

The integration of computational metabolomics tools such as molecular networking. MS2LDA and MolNetEnhancer workflows provided valuable insights into the chemical diversity of the wheat cultivars under study. Similar workflows have been successfully applied to annotate plant metabolites in two sorghum varieties, highlighting the utility of GNPS-based molecular networking in capturing cultivar-specific metabolite diversity [37]. Classical molecular networking facilitated the visualisation of the extracted wheat seed metabolomes as well as the putative annotation of the detected chemical signals. Additionally, feature-based molecular networks (FBMN) of the individual cultivars were also computed (links to the networking results are provided in the Supplementary Material). From the computed FBMN, the number of putatively annotated nodes matched to GNPS libraries for the individual cultivars were as follows: 260, 267, 257, 204 and 267 annotated nodes for the Elands, Gariep, Koonap, Matlabas and Senqu cultivars, respectively. Furthermore, expert (manual) validation of metabolite annotation was performed, and annotated metabolites are provided in Table S6. Moreover, contrary to classical MN, owing to the incorporation of MS1 information (i.e., retention time and relative abundance), FBMN also allowed for the identification of isomers featuring identical precursor masses but different retention times, which may have been incorrectly collapsed into a single node in classical MN [38]. Furthermore, enhanced (MolNetEnhancer) molecular networks were computed by integrating the classical MN workflow with MS2 latent Dirichlet allocation (MS2LDA) outputs for the individual cultivars, thus providing an overview of the chemical space in the extracted metabolomes and improving the chemical insights obtained from the outputs of the independent computational tools (Figure S1). The MS2LDA approach allows for the exploration of substructural diversity in the experimental fragmentation data by revealing patterns of co-occurring mass fragment peaks and/or neutral losses (referred to as Mass2Motifs, or MSM), which are often representative of molecular substructures or scaffolds [39,40,41].

As reflected by the Sunburst plot, flavonoids formed the largest group and contained 26% of the total number of putatively annotated metabolites in the extracted wheat seed metabolomes (Figure 3). According to [42], flavonoids are present in high levels in most plant grains and seeds. This class of secondary metabolites plays crucial roles in plant defence against pathogens as well as contributes to seed dormancy and maturation. Moreover, along with amino acids and their derivatives, flavonoids have been shown to be positively correlated to seed germination in rice [16]. The second largest class was lipids, consisting of 17% of the putatively annotated metabolites, followed by amino acids and HCA compounds, which comprised 12% and 9% of all annotated metabolites, respectively. Lipids play roles as a compact energy source in seeds for germination as well as serve as major biological membrane components; amino acids, on the other hand, serve as the major carrier of nitrogen delivered via the phloem vascular tissue to developing seeds [43,44].

### 4.1. The Primary Metabolism Charts in Dry Wheat Seeds

The differentiation of primary metabolites across the wheat cultivar seeds provides important insights into their physiological roles and potential agronomic implications. Amino acids, beyond their fundamental function as the main carriers of nitrogen (N), an important macronutrient in plant growth as well as in the balance of processing quality and yield in wheat production, also contribute to the synthesis of seed proteins which are essential for the wheat grain end-use quality [44,45,46]. Additionally, amino acids also contribute to stress reliance; for example, the elevated proline levels in Koonap and Matlabas seeds may indicate an inherent capacity (or metabolic predisposition) for greater stress tolerance, aligning with previous reports on the role of proline in cold and osmotic stress adaptation in plants [47]. Comparable associations between elevated proline and enhanced abiotic stress tolerance have been documented in barley seedlings [48] and rice varieties [49]. This suggests that metabolic composition could partially underlie cultivar-specific resilience traits. Sugars can serve either as an energy and carbon storage reserve for future biological processes or as protectants against stresses [50,51]. The relative abundance of sugars such as raffinose across the cultivar seeds may therefore reflect distinct metabolic strategies for balancing storage and stress adaptation during seed maturation. Lipid profiles also differed among the cultivars, highlighting their importance not only as storage reserves but also as contributors to seed vigour. The structural diversity of lipids—including phospholipids, glycolipids, and acylglycerols—underscores their dual role in forming cellular membranes and serving as a compact energy source for seed germination [43,52]. Differences in lipid composition could therefore influence both germination efficiency and seedling establishment.

Nucleic acid analogue levels provide insights into nucleotide metabolism, which further emphasises the metabolic preparedness of seeds for germination and early growth. That is, nucleotide metabolism and biosynthesis play crucial roles in providing building blocks for the synthesis of nucleic acids as well as serving as precursors for the synthesis of macromolecules such as polysaccharides, sucrose, and phospholipids. Moreover, nucleotide metabolism also plays an important role during early seedling development, seed germination and nutrient mobilisation in cotyledons of developing seedlings [53,54]. During purine nucleotide degradation, purine nucleotides are used as plant nutrient sources, during which the purine ring is oxidised and hydrolysed to carbon dioxide, ammonia and glyoxylate. Glyoxylate also forms part of the glyoxylate cycle, an important cycle which takes place in glyoxysomes and allows seeds to make use of lipids as an energy source for shoot formation during germination (i.e., it allows acetyl-CoA derived from the breakdown of storage lipids to be used for carbohydrate synthesis) [55,56]. Nucleic acid derivatives such as adenosine aid in cellular energy transfer by forming energy-carrying molecules, namely, adenosine diphosphate (ADP) and adenosine triphosphate (ATP); the latter provides the energy required to meet the metabolic demands of the seed germination stage [57].

Together, these observations demonstrate that cultivar-specific differences in amino acid, sugar, lipid, and nucleotide metabolism may underpin variation in stress tolerance, seed quality, and early growth potential. Such metabolic fingerprints provide a foundation for linking biochemical traits to breeding objectives focused on yield stability and stress resilience.

### 4.2. The Specialised Metabolism Charts in Dry Wheat Seeds

The accumulation of specialised metabolites across the wheat cultivar seeds highlights their potential roles in seed quality and stress resilience. Although the exact roles of the individual HCA compounds in wheat seed quality are not known, hydroxycinnamic acids are the most prevalent phenolic acids in maize and other grains, where they are covalently bonded to arabinoxylans of the cell wall hemicellulose, forming cross-linkages which serve as barriers to pathogen infection and insect herbivory in stored grain, strengthen and inhibit cell wall collapse during conditions of drought. Moreover, HCAs (especially ferulic acid) are also part of the major components of suberin, a cell wall barrier which protects plants against biotic and abiotic stresses [58,59]. The relatively high abundance of HCAs in Senqu and Elands seeds suggests these cultivars may possess enhanced physical and chemical defences against biotic and abiotic stressors. Flavonoids, which were also enriched in Senqu, Elands, and Koonap, are well-established contributors to seed maturation, dormancy, and longevity [42,60]. Beyond their defensive roles against pathogens, flavonoids in the seed coat are known to reduce water permeability and modulate oxygen availability, thereby delaying preharvest sprouting [60,61]. This trait is agriculturally significant, as preharvest sprouting contributes to yield loss and quality decline in wheat. The elevated flavonoid content in these cultivars may therefore contribute both to improved storability and to more robust germination performance. In particular, the high flavonoid levels observed in Koonap may underpin the cultivar’s potential resistance to fungal infection.

Other specialised metabolites also provide insights into seed function. Salicylic acid, detected in high levels in Elands seeds, is an established regulator of plant growth, respiration, and defence responses [62,63,64]. Our detection of salicylic acid also mirrors the role reported in common bean (*Phaseolus vulgaris*) seeds, where pretreatment with salicylic acid enhanced salt stress tolerance by boosting antioxidant defence [65]. Additionally, IAA and its derivatives were differentially distributed across the cultivars; this auxin has been shown to play crucial roles in processes such as gametogenesis, embryogenesis and growth of seedlings [62,66]. IAA has also been shown to play crucial roles in increasing seed membrane permeability for diffusion of water and regulation of activation of proteins and de novo synthesis of hydrolytic enzymes associated with hydration of seeds. Moreover, through improved protein synthesis and seed water content, and the promotion of cell division and elongation, the seed germination process is also favoured by the presence of IAA in seeds [67]. Together, these metabolites reflect how dry seeds retain biochemical signatures that may prime them for rapid activation during germination.

### 4.3. Biological Insights into Wheat Seed Metabolic Profiles

Although the mapped pathways above are not active in dry seeds, the aromatic amino acids produced via the Phe, Tyr and Trp biosynthesis pathway (Figure 6B), namely, tyrosine, phenylalanine and tryptophan, are involved in various important biological roles in plants, such as protein synthesis, energy metabolism and growth hormone regulation, and support the downstream biosynthesis of specialised plant defence metabolites in active seeds. For example, tyrosine serves as a precursor for the formation of diverse specialised metabolites such as benzylisoquinoline alkaloids, hydroxycinnamic acid amides and rosmarinic acid, which have various physiological roles in the plant seed life cycle [68]. Moreover, tyrosine has also been shown to have positive effects on plant growth and yield by serving as an energy, carbon and nitrogen source in plants [69]. Phenylalanine, on the other hand, serves as a precursor for the biosynthesis of flavonoids and isoflavonoids involved in defence mechanisms [70]. Similarly, in active seeds, tryptophan contributes to plant growth promotion by serving as a precursor for the biosynthesis of indole acetic acid (IAA), an essential plant growth and development regulating hormone (auxin) via the tryptophan metabolism pathway (Figure 6D). Additionally, tryptophan is also a precursor for other biologically important plant metabolites, including glucosinolates, phytoalexins, indoleamines and alkaloids [70,71,72].

In a metabolically active seed, these topological features of the network point to regulatory hubs that define the seed biochemistry and metabolism because the correlation matrix of metabolite pairs is a fingerprint of the enzymatic and regulatory reaction networks [73]. For instance, the amino acid tryptophan is clustered with the plant growth-promoting auxin indole acetic acid (IAA) and derivatives (Figure 7), which could point to (biosynthetic) regulatory mechanisms correlating tryptophan (a precursor) to IAA and derivatives. Thus, the results from both pathway and network analyses provide a biochemical description of wheat seed metabolism, revealing its key biological pathways and regulatory features which take place in active seeds. Although, to identify conditionally independent pairwise metabolic relationships, approaches (such as Gaussian graphical models and Bayesian networks) that enable decoupling direct from indirect variable associations could be used [74,75]. Importantly, these findings suggest that targeted breeding for metabolite traits, particularly phenolics and lipids, may enhance wheat resilience and seed quality under variable environmental conditions.

Taken together, the cultivar-related differences observed in the measured metabolome of wheat seeds point to cultivar-related nuances in seed quality, metabolic potential, growth and germination (Figure 8). As revealed by the relative quantification, the variations in metabolite levels across the different wheat cultivar seed samples spanned a wide range of chemical classes, with some cultivars being more enriched in certain classes compared to others. For example, the Senqu and Elands cultivars were rich in secondary metabolites such as HCAs and derivatives as well as flavonoids, which are mainly involved in plant defence against environmental stresses (i.e., biotic and abiotic stresses). Thus, it can be postulated that these two cultivars are more tolerant to stresses compared to cultivars such as Gariep and Matlabas cultivars, which had relatively low phenolic content. Moreover, flavonoids have also been positively correlated to seed germination [16]; therefore, the relatively high flavonoid content observed in seeds belonging to Senqu, Elands and Koonap cultivars positively influences seed germination. Matlabas and Gariep seeds were also found to have relatively low lipid contents (fatty acids, glycerolipids and oxylipins), which has been associated with the loss of seed viability and quality [76]. Thus, owing to their low lipid contents, it can be postulated that Gariep and Matlabas cultivar seeds have a relatively lower quality as compared to the other cultivar seeds. While Senqu, Elands and Koonap seeds can be hypothesised to be better in seed quality due to their high phenolics content, which can be translated to reduced sensitivity to stresses and positively influenced seed germination, which are two of the characteristics of high-quality seeds [10]. Furthermore, it is worth noting that the quality and viability of seeds is also influenced by the environment in which the seeds are grown, and some cultivars can perform better under certain environmental conditions but not others.

## 5. Conclusions

In this study, applying molecular networking workflows and functional analyses, we provide insight into the seed metabolomes of the wheat cultivars Senqu, Gariep, Matlabas, Elands and Koonap. The results showed that the metabolome of wheat seeds is characterised by a diverse array of metabolite classes, such as flavonoids, sugars, amino acids, lipids, HCA compounds and nucleic acid derivatives, among many others, which are involved in different physiological processes and functions in seeds, defining the quality and potential of the wheat seeds. The insights generated from this study have potential applications in terms of selections for seed nutrient and quality trait improvement in breeding programmes. Thus, this article is the first of its kind to comprehensively describe a metabolic chart of wheat seeds and to provide differential metabolic potentials of wheat seeds, with key features that can be used for seed quality assessment and improvement. Several limitations should be acknowledged. The metabolomic profiles presented here reflect dry seeds grown under specific conditions, and metabolite accumulation is known to vary with genotype–environment interactions. Despite these uncertainties, the insights generated here hold promise for practical applications. In particular, this study contributes to SDG 2 (Zero Hunger) by supporting the breeding of more nutritious and resilient wheat varieties, to SDG 12 (Responsible Consumption and Production) by enhancing seed quality and reducing crop losses, and to SDG 13 (Climate Action) by identifying metabolic traits that can guide the development of climate-resilient cultivars. Together, these contributions demonstrate how seed metabolomics can inform sustainable crop improvement strategies. Future studies such as targeted metabolomics analyses can be performed to validate the hypotheses postulated from this work. Moreover, other future studies can also include the integration of other omics approaches such as genomics to identify genes governing the key regulators of various desirable seed quality and nutrient content traits for wheat breeding programmes.

## Figures and Tables

**Table 1:** Characteristics of the wheat cultivars under study—namely Gariep, Koonap, Elands, Matlabas and Senqu—with indication to their susceptibility to aluminium toxicity and stripe rust [22].

Wheat Cultivar	Wheat Type	Resistance/Susceptibility
Gariep	Intermediate-type	Susceptible to both
Elands	Intermediate-type	Susceptible to both
Matlabas	Winter-type	Susceptible to both
Koonap	Intermediate-type	Resistant to both
Senqu	Intermediate-type	Susceptible to aluminium toxicity and resistant to stripe rust

## Data Availability

The datasets used during the current study are available from the corresponding author upon reasonable request.

## Supplementary Materials

The following supporting information can be downloaded at https://www.mdpi.com/article/10.3390/metabo15090603/s1, Table S1: Chromatographic separation conditions for targeted amino acids namely, serine, alanine, aspartic acid, threonine, proline, cysteine, valine and hydroxyproline; Table S2: Chromatographic separation conditions for targeted amino acids (i.e., tryptophan and tyrosine), hormones (i.e., indole-3-carboxyaldehyde, indole-3-carboxylic acid, 3-indoleacetic acid, methyl jasmonic acid and salicylic acid) as well as the nucleic acid derivative, N-hydroxyethylpthalamide; Table S3: Chromatographic separation conditions for targeted flavonoids namely, apigenin, luteolin, apigeninidin, luteolinidin, luteoside and vicenin-2; Table S4: Chromatographic separation conditions for targeted phenolics (i.e., avenanthramide A, avenanthramide B, ferulic acid, caffeic acid, hordenine) and D-fluorophenylalanine; Table S5: The multiple reaction monitoring MS (MRM-MS) analysis optimal conditions; Table S6: Putatively annotated metabolites (i.e., from FBMN) and targeted metabolites and the presence (**✓**) or absence (x) thereof across the cultivars under study; Table S7: MetaMapp identifiers for metabolic network analysis; Table S8: Metabolite classes, abbreviations and full names of all targeted and putatively annotated metabolites shown in the sunburst plot (Figure 3); Figure S1: Enhanced molecular networks.
