# Supplementary material for: Seed Metabolomic Landscape Reflecting Key Differential Metabolic Profiles Among Different Wheat Cultivars
Source: Metabolites. 2025 Sep 10;15(9):603. doi: 10.3390/metabo15090603 (PMC12471902; doi:10.3390/metabo15090603)
Supplement: Supplementary file 1 [file metabolites-15-00603-s001.zip › metabolites-3825147-supplementary.pdf]

Supplementary Materials

Seed metabolomic landscape reflecting key differential metabolic profiles among different wheat cultivars

Kgalaletso Othibeng [1], Lerato Nephali [1] and Fidele Tugizimana [1*]

Links for molecular networking jobs:

1. Classical MN (all cultivars): https://gnps.ucsd.edu/ProteoSAFe/status.jsp?task=831e0593f22c42a2850b90be5d31c974
2. FBMN (Elands): https://gnps.ucsd.edu/ProteoSAFe/status.jsp?task=0722ae929e09472a9d6c88787d37c745
3. FBMN (Koonap): https://gnps.ucsd.edu/ProteoSAFe/status.jsp?task=b29b8033fb0741d89fba29e0794b4cd3
4. FBMN (Gariep): https://gnps.ucsd.edu/ProteoSAFe/status.jsp?task=bb09a71769e24a5dbc4eccb5fa78abf0
5. FBMN (Matlabas): https://gnps.ucsd.edu/ProteoSAFe/status.jsp?task=30c79acb3c11466f8eb7ce4a095f6592
6. FBMN (Senqu): https://gnps.ucsd.edu/ProteoSAFe/status.jsp?task=94d6ad953191438fa8e9e8e4d951e1b9

Table S1: Chromatographic separation conditions for targeted amino acids namely, serine, alanine, aspartic acid, threonine, proline, cysteine, valine and hydroxyproline.

Time (min)	Solvent B Concentration (%)	Solvent A Concentration (%)
**1**	2	98
**2**	5	95
**3**	10	90
**5**	50	50
**7**	50	50
**7.1**	2	98
**10**	2	98
**10**	Stop	

Table S2: Chromatographic separation conditions for targeted amino acids (*i.e.,* tryptophan and tyrosine), hormones (*i.e.,* indole-3-carboxyaldehyde, indole-3-carboxylic acid, 3-indoleacetic acid, methyl jasmonic acid and salicylic acid) as well as the nucleic acid derivative, N-hydroxyethylpthalamide.

Time (min)	Solvent B Concentration (%)	Solvent A Concentration (%)
**3**	2	98
**4**	10	90
**6**	10	90
**20**	20	80
**30**	20	80
**33**	95	5
**38**	95	5
**40**	2	98
**43**	Stop	

Table S3: Chromatographic separation conditions for targeted flavonoids namely, apigenin, luteolin, apigeninidin, luteolinidin, luteoside and vicenin-2.

Time (min)	Solvent B Concentration (%)	Solvent A Concentration (%)
**2**	30	70
**12**	30	70
**25**	95	5
**30**	95	5
**31**	2	98
**37**	Stop	

Table S4: Chromatographic separation conditions for targeted phenolics (*i.e.,* avenanthramide A, avenanthramide B, ferulic acid, caffeic acid, hordenine) and D-fluorophenylalanine.

Time (min)	Solvent B Concentration (%)	Solvent A Concentration (%)
**1**	5	95
**25**	90	10
**26**	95	5
**29**	95	5
**30**	5	95
**35**	5	95
**35**	Stop	

Table S5: The multiple reaction monitoring MS (MRM-MS) analysis optimal conditions.

Compound name	Rt (min)	Ion mode	*m/z*	Transition	CE (eV)	Quadrupole 1 (Q1), V	Quadrupole 3 (Q3), V	Dwell time (msec)
**Amino acids**								
Serine	1.341	[M+H]	106.2	106.20>59.95<br>106.20>88.10	-13	-12	-10	17
Alanine	1.406	[M+H]	90.2	90.20>44.05<br>90.20>44.90	-13	-10	-15	17
Aspartic acid	1.41	[M+H]	134.05	134.05>74.10	-15	-10	-13	37
Threonine	1.409	[M+H]	120.2	120.20>56.05<br>120.20>74.10	-16	-13	-20	17
Proline	1.503	[M+H]	116.2	116.20>70.15<br>116.20>43.10	-18	-14	-11	17
Cysteine	1.317	[M+H]	241.2	241.20>151.90	-14	-12	-15	37
Valine	1.73	[M+H]	118.2	118.20>72.10<br>118.20>55.05	-12	-14	-12	17
Hydroxyproline	1.431	[M+H]	132.2	132.20>86.05<br>132.20>68.05	-15	-10	-15	17
Tryptophan	8.129	[M+H]	205	205.20>188.05<br>205.20>146.10	-10			100
Tyrosine	3.772	[M+H]	182	182>136.10	-29			100
D-Fluorophenylalanine	5.304	[M+H]	184	184.00>138.15<br>184.00>118.15<br>184.00>91.15	-14	-13	-25	100
**Phenolics**								
Apigenin	14.989	[M+H]	271	271.00>253.10<br>271.00>239.20<br>271.00>153.00	-9	-12	-26	100
Luteolin	14.38	[M+H]	287	287.00>269.20<br>287.00>246.10<br>287.00>153.05	-8	-23	-19	100

Apigeninidin	15.226	[M+H]	256	256.00>57.10<br>256.00>215.15<br>256.00>88.05	-24	-18	-19	100
Luteolinidin	11.749	[M+H]	272	272.00>229.50<br>272.00>254.15<br>272.00>231.05	-35	-10	-13	100
Luteoside	10.022	[M+H]	449	449.00>287.15<br>449.00>417.15<br>449.00>153.10	-21	-11	-19	100
Vicenin 2	4.357	[M+H]	595	595.00>324.90<br>595.00>379.20<br>595.00>475.25	-35	-22	-22	100
Avenanthramide A	17.081	[M+H]	300.2877	330.2877>330.28	-5	-22	-21	100
Avenanthramide B	17.493	[M+H]	330.3078	330.3078>330.30	-6	-12	-23	100
Ferulic acid	12.418	[M+H]	195.1878	195.1878>195.18	-8	-14	-22	100
Caffeic acid	9.745	[M+H]	181.678	181.1678>181.16	-8	-13	-13	100
Hordenine	4.872	[M+H]	166.2378	166.2378>166.23	-10	-12	-30	100
**Nucleic acid derivative**								
N-hydroxyethylpthalamide	30.075	[M+H]	192.15	192.15>192.15	-10			100
**Hormones**								
Indole-3-carboxyaldehyde	16.607	[M-H]	144.35	144.35>144.35	28			100
Indole-3-carboxylicacid	21.953	[M-H]	160.05	160.05>160.05	15			100
IAA	26.812	[M+H]	176.1	176.10>130.10<br>176.10>77.20<br>176.10>103.10	-15	-20	-20	65.6
MeJA	7.917	[M+H]	225.2	225.20>151.15<br>225.20>133.15<br>225.20>147.15	-15	-20	-20	65.6
SA	22.598	[M-H]	137	137.00>92.95<br>137.00>65.00	15	20	20	65.6

Table S6: Putatively annotated metabolites (*i.e.,* from FBMN) and targeted metabolites and the presence (✓) or absence (x) thereof across the cultivars under study.

Metabolite name	m/z	Rt	Adduct	Formula	Metabolite class	Elands	Gariep	Koonap	Matlabas	Senqu
**1-Hexadecanoyl-sn-glycero-3-phospho-(1'-sn-glycerol)**	483.4471	19.622	[M-H]	C22H45O9P	Lipid	✓	✓	✓	✓	✓
**isorhamnetin-3-O-rutinoside**	623.2359	9.419	[M-H]	C28H32O16	Flavonoid	✓	x	x	x	x
**Melitriose/ Raffinose**	503.1687	1.124	[M-H]	C18H32O16	Sugar	✓	✓	✓	✓	✓
**(10E,15E)-9,12,13-trihydroxyoctadeca-10,15-dienoic acid (9,12,13-TriHODE)**	327.222	15.953	[M-H]	C18H32O5	Lipid	✓	✓	x	✓	x
**(2S,3S,4S,5R,6R)-6-(3-benzoyloxy-2-hydroxypropoxy)-3,4,5-trihydroxyoxane-2-carboxylic acid**	371.2395	7.447	[M-H]	C16H20O10	Carbohydrate derivative	✓	x	x	x	x
**(4E)-7-acetyloxy-6-hydroxy-2-methyl-10-oxo-2,3,6,7,8,9-hexahydrooxecin-3-yl] (E)-but-2-enoate**	325.1884	18.753; 19.157; 19.321; 19.494	[M-H]	C16H22O7	Carbohydrate derivative	✓	✓	x	✓	✓
**12, 13-DiHOME**	313.3156	17.199	[M-H]	C18H32O4	Lipid	x	✓	✓	✓	✓
**13-HODE**	295.1829	19.467	[M-H]	C18H32O3	Lipid	x	✓	x	✓	x
**13-HOTrE**	293.2999	15.624	[M-H]	C18H30O3	Lipid	✓	✓	✓	✓	✓
**13-KODE**	293.2159	16.23; 16.534	[M-H]	C18H30O3	Lipid	x	x	✓	✓	✓
**13-Labdene-2,8,15-triol**	323.1722	18.578	[M-H]	C20H36O3	Lipid	x	x	✓	x	x
**1-Oleoylglycerophosphoinositol**	597.3121	19.275	[M-H]	C27H51OO12P	Lipid		✓	✓	✓	✓
**1-palmitoylglycerophosphoinositol**	571.2969	18.247	[M-H]	C25H49O12P	Lipid	x	x	x	✓	x
**2,4-dihydroxyheptadec-16-yn-1-yl acetate**	325.2428	21.935	[M-H]	C19H34O4	Fatty alcohol	✓	✓	✓	✓	✓

**3-(Benzoyloxy)-2-hydroxypropyl beta-D-glucopyranosiduronic acid**	371.1032	7.452	[M-H]	C16H20O10	Phenolic	x	✓	✓	✓	x
**3,4-Dicaffeoyl quinic acid**	515.1259	9.498	[M-H]	C25H24O12	HCA Compound	x	✓	x	x	x
**3,4-Dihydroxyflavone**	253.0747	6.887	[M-H]	C15H10O4	Flavonoid	x	x	x	x	✓
**3-Feruloylquinic acid**	367.1086	6.555	[M-H]	C17H20O9	HCA Compound	x	x	x	✓	x
**3-Indolelactic acid**	204.0694	8.237	[M-H]	C11H11NO3	Hormone	✓	✓	x	x	x
**3-O-Feruloylsucrose**	517.1631	6.49	[M-H]	C22H30O14	HCA Compound	✓	✓	✓	x	x
**4,5-Dicaffeoyl quinic acid**	515.1274	9.489	[M-H]	C25H24O12	HCA Compound	✓	x	x	x	x
**4-Caffeoylquinic acid**	353.0923	6.332	[M-H]	C16H18O9	HCA Compound	x	x	x	✓	✓
**5,6,2'-Trimethoxyflavone**	311.2453	18.259	[M-H]	C18H16O5	Flavonoid	✓	✓	✓	✓	✓
**5,8,11-trihydroxyoctadec-9-enoic acid**	329.2377	14.503; 14.393; 14.695	[M-H]	C18H34O5	Lipid	x	x	✓	✓	✓
**9-(2,3-dihydroxypropoxy)-9-oxononanoic acid**	261.1376	8.227; 9.138	[M-H]	C12H22O6	Lipid	x	x	✓	x	x
**9, 10-DiHOME**	313.3152	17.453	[M-H]	C18H32O4	Lipid	✓	✓	✓	✓	✓
**9,12,13-TriHODE**	327.3199	12.762	[M-H]	C18H32O5	Lipid	x	x	✓	✓	✓
**9-KODE**	293.179	16.351	[M-H]	C18H30O3	Lipid	✓	✓	x	✓	x
**Acanthoside B**	579.2163	9.527	[M-H]	C28H36O13	Sugar	x	✓	✓	x	x
**Adenosine (conjugated to something)**	312.0988	1.778	[M-H]	C10H13N5O5	Nucleic acid derivative	x	x	✓	✓	✓
**Apigenin 6-C-glucoside 8-C-arabinoside**	563.1478	7.134	[M-H]	C26H28O14	Flavonoid	x	x	x	✓	x

**Apigenin 6-C-I<<-L-arabinopyranosyl-8-C-EC-D-xylopyranoside**	533.137 3	8.79	[M-H]	C25H26O13	Flavonoid	x	x	✓	x	x
**Arillatose B**	517.163	6.303	[M-H]	C22H30O14	Phenylpropanoi d	x	x	✓	✓	✓
**auraptene**	297.156 1	16.969	[M-H]	C19H22O3	Phenolic acid	x	x	✓	x	✓
**Azelaic acid**	187.277 3	9.405	[M-H]	C9H16O4	Organic acid	✓	x	✓	✓	x
**Canrenone**	339.204 1	20.715	[M-H]	C22H28O3	Steroid lactone	✓	✓	✓	✓	✓
**Chlorogenic acid**	353.092 4	6.321	[M-H]	C16H18O9	HCA Compound	✓	x	✓	x	x
**Chrysoeriol 8-C-glucoside**	461.115 4	8.899	[M-H]	C22H22O11	Flavonoid	✓	x	x	x	x
**Citric acid**	191.078 8	1.183	[M-H]	C6H8O7	Organic acid	x	✓	x	✓	✓
**Corymboside**	563.261 1	7.915	[M-H]	C26H28O14	Flavonoid	✓	✓	✓	x	✓
**Demethoxycentaureidin 7-O-rutinoside**	637.186 3	9.57	[M-H]	C29H34O16	Flavonoid	✓	✓	x	x	✓
**Deoxyguanosine**	266.093 2	2.213	[M-H]	C10H13N5O4	Nucleic acid derivative	x	✓	x	x	x
**DGMG 18:2**	675.368 8	17.638	[M-H]	C33H56O14	Lipid	x	x	x	x	✓
**Dicoumaroyl Spermidine**	436.229 4	8.831	[M-H]	C25H31N3O4	Alkaloid	✓	x	x	x	x
**Diferuloyl glycerol**	443.099 2	13.436	[M-H]	C23H23O9	HCA Compound	✓	✓	✓	x	✓
**DIMBOA derivative**	534.153 3	6.454	[M-H]	C21H29NO15	Lipid	x	x	x	✓	x
**Endocrocin**	313.039 3	12.121	[M-H]	C16H10O7	Anthraquinones	✓	x	x	x	x
**Feruloyl hexoside**	355.108 2	6.754	[M-H]	C16H20O9	HCA derivative	x	✓	x	x	x

**Feruloylquinic acid**	367.108	6.547	[M-H]	C17H20O9	HCA Compound	✓	x	x	x	x
**Fraxin**	369.0878	6.784	[M-H]	C16H18O10	Glucoside	✓	x	x	x	✓
**Gardnerine**	323.1732	18.202	[M-H]	C20H24N2O2	Indole alkaloids	✓	x	x	x	x
**Guanosine**	282.088	1.844	[M-H]	C10H13N5O5	Nucleic acid derivative	✓	✓	✓	x	✓
**Isoquercitirin (same as isoquercetin)**	463.0942	8.634	[M-H]	C21H20O12	Flavonoid	x	✓	✓	x	x
**Isoschaftoside**	563.1489	7.76	[M-H]	C26H28O14	Flavonoid	✓	✓	✓	✓	✓
**Isovitexin**	431.1042	8.749	[M-H]	C21H20O10	Flavonoid	x	✓	x	x	x
**Juniperoside III**	311.2054	20.264	[M-H]	C15H20O7	Sugar	x	x	✓	✓	✓
**Maltotriose**	503.1682	1.634	[M-H]	C18H32O16	Sugar	✓	✓	✓	x	x
**Melibiose (ADDUCT)**	387.1193	1.573	[M+2Na-H]	C12H22O11	Sugar	✓	✓	✓	✓	✓
**methyl (2E,4E,8E)-7,13-dihydroxy-4,8,12-trimethyltetradeca-2,4,8-trienoate**	309.1566	17.134	[M-H]	C18H30O4	Lipid	✓	x	✓	x	✓
**MGMG 18:2**	561.5116	19.812	[M+HCOO-H]	C27H48O9	Lipid	✓	✓	✓	✓	✓
**Octanedioic acid**	173.0841	7.892	[M-H]	C8H14O4	Lipid	x	x	x	✓	x
**Palatinose**	377.0906	1.476	[M+Cl-H]	C12H22O11	Sugar	x	x	x	✓	x
**Pinolidoxin**	337.1874	19.324	[M-H]	C18H26O6	Oxocin	x	x	x	x	✓
**Pyrenophorol**	311.1723	18.061	[M-H]	C16H24O6	Macrolide	x	✓	x	x	✓
**Quercetin-3-(2-glucosylrhamnoside)**	609.1534	8.409	[M-H]	C27H30O16	Flavonoid	x	✓	x	x	x

**Raffinose**	539.1461	1.12	[M+Cl-H]	C18H32O16	Sugar	✓	✓	✓	✓	✓
**Rutin**	609.1534	8.39	[M-H]	C27H30O16	Flavonoid	✓	x	x	x	✓
**Scoparin**	461.1144	8.914	[M-H]	C22H22O11	Flavonoid	x	x	x	x	✓
**Scutellarioside II**	507.1128	10.452	[M-H]	C24H28O12	HCA derivative	✓	x	x	x	x
**Stachyose**	665.2239	1.162	[M-H]	C24H24O21	Sugar	x	✓	✓	✓	✓
**sucrose**	377.1256	0.769	[M+Cl-H]	C12H22O11	Sugar	✓	✓	✓	x	✓
**trans-caffeic acid**	179.0371	6.5	[M-H]	C9H8O4	HCA derivative	x	x	x	✓	✓
**Trehalose**	341.1133	3.66; 4.889	[M-H]	C12H22O11	Sugar	x	x	✓	✓	✓
**Tricin**	329.0708	12.791	[M-H]	C17H14O7	Flavonoid	✓	✓	✓	x	✓
**Tricin-7-glucoside**	491.1257	9.89	[M-H]	C23H24O12	Flavonoid		✓	x	✓	✓
**trihydroxyoctadec-9-enoic acid**	329.2379	15.798	[M-H]	C18H34O5	Lipid	✓	✓	x	x	x
**UDP-N-acetylglucosamine**	606.0829	1.279	[M-H]	C17H27N3O17P2	Nucleic acid derivative	x	x	x	✓	x
**Vicenin-2**	593.1596	7.062; 7.179	[M-H]	C27H30O15	Flavonoid	✓	✓	✓	✓	✓
**Violanthin**	577.1636	8.244	[M-H]	C27H30O14	Flavonoid	x	✓	x	✓	✓
**Vitexin**	431.1046	8.742	[M-H]	C21H20O10	Flavonoid	✓	x	x	✓	✓
**xanthosine**	283.0719	2.61	[M-H]	C10H12N4O6	Nucleic acid derivative	x	x	x	✓	x
**Serine**	106.2	1.341	[M+H]	C3H7NO3	Amino acid	✓	x	✓	x	✓
**Alanine**	90.2	1.406	[M+H]	C3H7NO2	Amino acid	✓	✓	✓	✓	✓
**Aspartic acid**	134.05	1.41	[M+H]	C4H7NO4	Amino acid	✓	✓	✓	✓	✓

**Threonine**	120.2	1.409	[M+H]	C4H9NO3	Amino acid	✓	✓	✓	✓	✓
**Proline**	116.2	1.503	[M+H]	C5H9NO2	Amino acid	✓	✓	✓	✓	✓
**Cysteine**	241.2	1.317	[M+H]	C3H7NO2S	Amino acid	✓	✓	✓	✓	✓
**Valine**	118.2	1.73	[M+H]	C5H11NO2	Amino acid	✓	✓	✓	✓	✓
**Hydroxyproline**	132.2	1.431	[M+H]	C5H9NO3	Amino acid	✓	✓	✓	✓	✓
**Tryptophan**	205	8.129	[M+H]	C11H12N2O2	Amino acid	✓	✓	✓	✓	✓
**Tyrosine**	182	3.772	[M+H]	C9H11NO3	Amino acid	✓	✓	✓	✓	✓
**D-Fluorophenylalanine**	184	5.304	[M+H]	C9H10FNO2	Amino acid	✓	✓	✓	✓	✓
**Apigenin**	271	14.989	[M+H]	C15H10O5	Flavonoid	✓	✓	✓	✓	✓
**Luteolin**	287	14.38	[M+H]	C15H10O6	Flavonoid	✓	✓	✓	✓	✓
**Apigeninidin**	256	15.226	[M+H]	C15H11ClO4	Flavonoid	✓	✓	✓	✓	✓
**Luteolinidin**	272	11.749	[M+H]	C15H11O5	Flavonoid	✓	✓	✓	✓	✓
**Luteoside**	449	10.022	[M+H]	C28H32O16	Flavonoid	✓	✓	✓	✓	✓
**Apigenitrin**	433	15.042	[M+H]		Flavonoid	✓	✓	✓	✓	✓
**Vicenin 2**	595	4.357	[M+H]	C27H30O15	Flavonoid	x	x	✓	✓	✓
**Avenanthramide A**	300.287 7	17.081	[M+H]	C16H13NO5	Phenolic alkaloid	✓	✓	✓	✓	✓
**Avenanthramide B**	330.307 8	17.493	[M+H]	C17H15NO6	Phenolic alkaloid	✓	✓	✓	✓	✓
**Ferulic acid**	195.187 8	12.418	[M+H]	C10H10O4	HCA derivative	✓	✓	✓	✓	✓
**Caffeic acid**	181.678	9.745	[M+H]	C9H8O4	HCA derivative	✓	✓	✓	✓	✓
**Hordenine**	166.237 8	4.872	[M+H]	C10H15NO	Phenolic alkaloid	✓	✓	✓	✓	✓
**N-hydroxyethylpthalamide**	192.15	30.075	[M+H]	C10H9NO3	Nucleic acid derivative	✓	✓	✓	✓	✓
**Indole-3-carboxyaldehyde**	144.35	16.607	[M-H]	C9H7NO	Hormone	✓	x	✓	✓	x
**Indole-3-carboxylicacid**	160.05	21.953	[M-H]	C9H7NO2	Hormone	✓	✓	✓	✓	✓
**3-indoleacetic acid (IAA)**	176.1	26.812	[M+H]	C10H9NO2	Hormone	✓	✓	✓	✓	✓
**Methyl jasmonic acid (MeJA)**	225.2	7.917	[M+H]	C13H20O3	Hormone	✓	✓	✓	✓	✓
**Salicylic acid (SA)**	137	22.598	[M-H]	C7H6O3	Hormone	✓	x	x	x	x

Table S7: MetaMapp identifiers for metabolic network analysis

PubChem_ID	KEGG_ID	SMILES	Compound_Name	Condition_A_pvalue	Condition_A_foldchange
338	C00805	C1=CC=C(C(=C1)C(=O)O)O	SA	1.08E-08	1.2
802	C00954	C1=CC=C2C(=C1)C(=CN2)CC(=O)O	IAA	1.48E-07	1.0625
1198	C00311	C(C(C(C(=O)O)O)C(=O)O)C(=O)O	Citric acid	0.028342	0.691042
2266	C08261	C(CCCC(=O)O)CCCC(=O)O	Azelaic acid	6.29E-09	2.43425
4654		C1=CC(=CC=C1CC(C(=O)O)N)F	D-Fluorophenylalanine	0.156464	0.965727
5862	C00097	C(C(C(=O)O)N)S	Cysteine	0.000277	3.36608
5950	C00041	CC(C(=O)O)N	Alanine	1.43E-07	3.19637
5951	C00716	C(C(C(=O)O)N)O	Serine	2.03E-08	1.5
5960	C00049	C(C(C(=O)O)N)C(=O)O	Aspartic acid	0.000147	0.792112
5988	C00089	C(C1C(C(C(C(O1)OC2(C(C(C(O2)CO)O)O)CO)O)O)O)O	Sucrose	4.44E-13	2.1571
6057	C00082	C1=CC(=CC=C1CC(C(=O)O)N)O	Tyrosine	0.181714	2.42954
6287	C00183	CC(C)C(C(=O)O)N	Valine	3.58E-08	0.112141
6288	C00188	CC(C(C(=O)O)N)O	Threonine	6.61E-06	1.95923
6305	C00806	C1=CC=C2C(=C1)C(=CN2)CC(C(=O)O)N	Tryptophan	0.040655	0.871098
7427	C01083	C(C1C(C(C(C(O1)OC2C(C(C(C(O2)CO)O)O)O)O)O)O)O	Trehalose	6.12E-14	2.2043

10256	C08493	C1=CC=C2C(=C1)C(=CN2)C=O	Indole-3-carboxyaldehyde	0.004517	21.8592
10457	C08278	C(CCCC(=O)O)CCC(=O)O	Suberic acid	1.22E-14	4.66863
13789	D03363	CC12CCC(=O)C=C1C=CC3C2CCC4(C3CCC45CCC(=O)O5)C	Canrenone	0.233917	1.22804
60961	C00212	C1=NC(=C2C(=N1)N(C=N2)[C@H]3[C@@H]([C@@H]([C@H](O3)CO)O)O)N	Adenosine	4.13E-11	2.57231
68313	C06199	CN(C)CCC1=CC=C(C=C1)O	Hordenine	5.64E-05	0.309343
69867	C19837	C1=CC=C2C(=C1)C(=CN2)C(=O)O	Indole-3-carboxylicacid	0.003062	0.538064
77499		C1=CC=C2C(=C1)C(=O)N(C2=O)CCO	N-hydroxyethylpthalImide	3.10E-13	0.24411
92904	C02043	C1=CC=C2C(=C1)C(=CN2)CC(C(=O)O)O	3-Indolelactic acid	4.37E-11	3.23865
145726		C1=CC=C2C(=C1)C(=O)C=C(O2)C3=CC(=C(C=C3)O)O	3,4-Dihydroxyflavone	2.79E-06	9.2682
145742	C00148	C1CC(NC1)C(=O)O	Proline	2.01E-12	2.29095
159360		C1=CC(=CC=C1C2=[O+]C3=CC(=CC(=C3C=C2)O)O)O.[Cl-]	Apigeninidin	0.073447	1.00937
439242	C00492	C(C1C(C(C(C(O1)OCC2C(C(C(C(O2)OC3(C(C(C(O3)CO)O)O)CO)O)O)O)O)O)O)O	Melitriose	7.92E-06	1.6864
439559	C01742	C(C1C(C(C(C(O1)OCC2C(C(C(O2)(CO)O)O)O)O)O)O)O	Palatinose	4.02E-12	2.34932
440658	C05402	C(C1C(C(C(C(O1)OCC2C(C(C(C(O2)O)O)O)O)O)O)O)O	Melibiose	5.48E-15	2.43369
441701	C08652	C1=CC(=C(C=C1C2=[O+]C3=CC(=CC(=C3C=C2)O)O)O)O	Luteolinidin	0.072653	1.00903
442664	C10195	C1=CC(=CC=C1C2=CC(=O)C3=C(C(=C(C(=C3O2)C4C(C(C(C(O4)CO)O)O)O)O)C5C(C(C(C(O5)CO)O)O)O)O)O	Vicenin-2	2.11E-11	2.31991
442665	C10196	CC1C(C(C(C(O1)C2=C3C(=C(C(=C2O)C4C(C(C(C(O4)CO)O)O)O)O)C(=O)C=C(O3)C5=CC=C(C=C5)O)O)O)O	Violanthin	5.34E-12	2.628
443024	C10890	COC1=CC(=CC(=C1O)OC)C2C3COC(C3CO2)C4=CC(=C(C(=C4)OC)OC5C(C(C(C(O5)CO)O)O)O)OC	Acanthoside B	3.77E-05	0.518583

445675	C00043	CC(=O)NC1C(C(C(OC1OP(=O)(O)OP(=O)(O)OCC2C(C(C(O2)N3C=CC(=O)NC3=O)O)O)CO)O)O	UDP-N-acetylglucosamine	0.004746	6.89557
445858	C01494	COC1=C(C=CC(=C1)C=CC(=O)O)O	Ferulic acid	0.000861	1.53046
689043	C01197	C1=CC(=C(C=C1C=CC(=O)O)O)O	Caffeic acid	0.40205	1.55283
1550607		CC(=CCCC(=CCOC1=CC2=C(C=C1)C=CC(=O)O2)C)C	aurapten	0.000389	2.53154
1794427	C00852	C1C(C(C(CC1(C(=O)O)O)OC(=O)C=CC2=CC(=C(C=C2)O)O)O)O	4-Caffeoylquinic acid	7.07E-05	2.42372
3084995		C1C(C(C(C(O1)C2=C(C(=C3C(=C2O)C(=O)C=C(O3)C4=CC=C(C=C4)O)C5C(C(C(C(O5)CO)O)O)O)O)O)O)O	Isoschaftoside	0.010496	1.58761
3952079		CC(=O)OCC(CC(CCCCCCCCCCCC#C)O)O	2,4-dihydroxyheptadec-16-yn-1-yl acetate	0.022505	1.75214
5273568		COC1=C(C(=C2C(=C1)C=CC(=O)O2)OC3C(C(C(C(O3)CO)O)O)O)O	Fraxin(Fraxoside)	0.015588	1.88693
5280441	C01460	C1=CC(=CC=C1C2=CC(=O)C3=C(O2)C(=C(C=C3O)O)C4C(C(C(C(O4)CO)O)O)O)O	Vitexin	3.76E-08	3.04844
5280443	C01477	C1=CC(=CC=C1C2=CC(=O)C3=C(C=C(C=C3O2)O)O)O	Apigenin	0.119721	1.00722
5280445	C01514	C1=CC(=C(C=C1C2=CC(=O)C3=C(C=C(C=C3O2)O)O)O)O	Luteolin	0.003529	1.01155
5280804	C05623	C1=CC(=C(C=C1C2=C(C(=O)C3=C(C=C(C=C3O2)O)O)OC4C(C(C(C(O4)CO)O)O)O)O)O	isoquercetin	6.50E-10	17.6983
5280805	C05625	CC1C(C(C(C(O1)OCC2C(C(C(C(O2)OC3=C(OC4=CC(=CC(=C4C3=O)O)O)C5=CC(=C(C=C5)O)O)O)O)O)O)O)O	Rutin	6.95E-05	5.95564
5281157	C08472	C1=CC(=CC=C1C=CC(=O)NC2=C(C=C(C=C2)O)C(=O)O)O	Avenanthramide A	7.07E-06	6.60843
5281169	C08503	CCCC1C(C(C=CCCC(C(=O)O1)OC(=O)C=CC=CC)O)O	Pinolidoxin	0.890043	1.07842
5281702	C10193	COC1=CC(=CC(=C1O)OC)C2=CC(=O)C3=C(C=C(C=C3O2)O)O	Tricin	0.009631	3.29679
5282947	C14762	CCCCCC(C=CC=CCCCCCCCC(=O)O)O	13-HODE	0.026423	0.665869
5312876		CCC=CCC(C(C=CC(CCCCCCCC(=O)O)O)O)O	9,12,13-TriHODE	4.66E-08	2.93193

5319693	C11512	CCC=CCC1C(CCC1=O)CC(=O)OC	MEJA	1.03E-06	0.77961
5322022		COC1=CC(=CC(=C1O)OC)C2=CC(=O)C3=C(C=C(C=C3O2)OC4C(C(C(C(O4)CO)O)O)O)O)O	Tricin-7-glucoside	4.17E-06	1.81905
5481663		CC1C(C(C(C(O1)OCC2C(C(C(C(O2)OC3=C(OC4=CC(=CC(=C4C3=O)O)O)C5=CC(=C(C=C5)O)OC)O)O)O)O)O)O	isorhamnetin-3-O-rutinoside	2.62E-07	2.50681
6326020		COC1=C(C=CC(=C1)C=CC(=O)OC2C(C(OC2(CO)OC3C(C(C(C(O3)CO)O)O)O)CO)O)O	3-O-Feruloylsucrose	7.20E-10	2.24786
6445140		CC=C1CN2C3CC1C(C2CC4=C3NC5=C4C=CC(=C5)OC)CO	Gardnerine	0.014525	2.12663
9839084	C14766	CCCCCC=CC=CC(=O)CCCCCCCC(=O)O	9-KODE	0.00601	1.21264
10087955		COC1=C(C=CC(=C1)C=CC(=O)NC2=C(C=C(C=C2)O)C(=O)O)O	Avenanthramide B	0.248373	4.93355
10133609	C02572	COC1=C(C=CC(=C1)C=CC(=O)OC2CC(CC(C2O)O)(C(=O)O)O)O	Feruloylquinic acid	3.36E-09	2.46801
10469728		CCC=CCC(C=CC=CCCCCCCCC(=O)O)O	13-HOTrE I	0.005769	1.21977
10935870		CC1CCC(C=CC(=O)OC(CCC(C=CC(=O)O1)O)C)O	Pyrenophorol	0.012806	1.99338
13644660		C1C(C(C(C(O1)C2=C(C(=C3C(=C2O)C(=O)C=C(O3)C4=CC=C(C=C4)O)C5C(C(C(C(O5)CO)O)O)O)O)O)O)O)O	Corymboside	1.09E-07	12.0004
13887346	C10445	C1C(C(C(CC1(C(=O)O)O)OC(=O)C=CC2=CC(=C(C=C2)O)O)OC(=O)C=CC3=CC(=C(C=C3)O)O)O	4,5-Dicaffeoyl quinic acid	0.000636	6.25516
13962927		COC1=C(C=CC(=C1)C=CC(=O)OC2C(C(C(C(O2)CO)O)O)O)O	Feruloyl hexoside	0.952834	0.991818
14427338		C1C(C(C(C(O1)C2=C(C(=C3C(=C2O)C(=O)C=C(O3)C4=CC=C(C=C4)O)C5C(C(C(CO5)O)O)O)O)O)O)O	Apigenin-6-C-I<<-L-arabinopyranosyl-8-C-EC-D-xylopyranoside	7.82E-11	2.34452
14484690		COC1=C(C2=C(C=C1)OC(=CC2=O)C3=CC=CC=C3OC)OC	5,6,2'-Trimethoxyflavone	1.56E-09	5.29443
15236084		CCCCCC=CCC=CCCCCCCCC(=O)OCC(COC1C(C(C(C(O1)CO)O)O)O)O	MGMG 18:2	0.038467	0.606201
15270795		C1=CC(=CC=C1C=CC(=O)NCCCCNCCCNC(=O)C=CC2=CC=C(C=C2)O)O	Dicoumaroyl Spermidine	6.39E-06	5.9862
20106119		CC1C(C(C(C(O1)OCC2C(C(C(C(O2)OC3=C(C(=C4C(=C3)OC(=CC4=O)C5=CC(=C(C=C5)O)OC)O)OC)O)O)O)O)O)O	Demethoxycentaureidin 7-O-rutinoside	9.14E-05	1.35537

42607483		CCCCCCCCCCCCCCCC(=O)OCC(COP(=O)(O)OCC(CO)O)O	1-Hexadecanoyl-sn-glycero-3-phospho-(1'-sn-glycerol)	0.117249	0.653737
44258170		COC1=C(C=CC(=C1)C2=CC(=O)C3=C(O2)C(=C(C=C3O)O)C4C(C(C(C(O4)CO)O)O)O)O	Chrysoeriol 8-C-glucoside (scoparin)	2.39E-09	0.203624
45359333		CC=CC(=O)OC1C=CC(C(CCC(=O)OC1C)OC(=O)C)O	(4E)-7-acetyloxy-6-hydroxy-2-methyl-10-oxo-2,3,6,7,8,9-hexahydrooxecin-3-yl] (E)-but-2-enoate	0.010045	2.07122
45783079		C1=CC=C(C=C1)C(=O)OCC(COC2C(C(C(C(O2)C(=O)O)O)O)O)O	(2S,3S,4S,5R,6R)-6-(3-benzoyloxy-2-hydroxypropoxy)-3,4,5-trihydroxyoxane-2-carboxylic acid	0.101331	0.76431
45783154		C(CCCC(=O)O)CCCC(=O)OCC(CO)O	9-(2,3-dihydroxypropoxy)-9-oxononanoic acid I	8.55E-15	4.05663
53941874		CC(CCCC(C(CC=CCCCCCCCC(=O)O)O)O)O	trihydroxyoctadec-9-enoic acid	4.06E-10	2.16507
56773932		CC(CCC=C(C)C(CC=C(C)C=CC(=O)OC)O)C(C)O	methyl (2E,4E,8E)-7,13-dihydroxy-4,8,12-trimethyltetradeca-2,4,8-trienoate	0.003104	5.31784
56776330		CC(=CCO)CCC1C(CCC2C1(CC(CC2(C)C)O)C)(C)O	13-Labdene-2,8,15-triol	0.292461	1.36437
56776385		CC1C(C(C(=CC=C1)OC2C(C(C(C(O2)CO)O)O)O)C(=O)C	Juniperoside III	0.112926	1.19245
71296206		CCCCCCCCCCCCCCCC(=O)OCC(COP(=O)([O-])OC1C(C(C(C(C1O)O)O)O)O)O	1-palmitoylglycerophosphoinositol	0.081608	0.771523
72188972		CC1C(C(C(C(O1)OC2=CC(=C3C(=C2)OC(=C(C3=O)OC4C(C(C(C(O4)CO)O)O)O)C5=CC(=C(C=C5)O)OC)O)O)O)O	Luteoside	0.016657	0.996783

76967301		COC1=C(C=CC(=C1)C=CC(=O)OCC(CO)OC(=O)C=CC2=CC(=C(C=C2)O)OC)O	Diferuloyl glycerol	7.68E-05	0.289597
86289645		CCCCCCCCC=CCCCCCCCC(=O)OCC(COP(=O)(O)OC1C(C(C(C(C1O)O)O)O)O)O	1-Oleoylglycerophosphoinositol	0.081159	1.42095
1.26E+08		CCCCCC=CCC(C(CCCCCCCC(=O)O)O)O	9, 10-DiHOME	0.000326	1.18088
1.35E+08	C00330	C1C(C(OC1N2C=NC3=C2N=C(NC3=O)N)CO)O	Deoxyguanosine	0.622879	1.0559
1.35E+08	C00387	C1=NC2=C(N1C3C(C(C(O3)CO)O)O)N=C(NC2=O)N	Guanosine	3.80E-07	1.79507
1.39E+08		CCC=CCC=CCC=CCCCCCCCC(=O)OCC(COC1C(C(C(C(O1)COC2C(C(C(C(O2)CO)O)O)O)O)O)O)O	DGMG 18:3	0.00631	1.9046
1.39E+08		COC1=CC2=C(C=C1)N(C(=O)C(O2)OC3C(C(C(C(O3)CO)O)O)OC4C(C(C(C(O4)CO)O)O)O)O	DIMBOA + O-Hex-Hex	9.61E-17	10.7274
1.63E+08		CCCCCC(C(CC=CCCCCCCCC(=O)O)O)O	12, 13-DiHOME	0.193949	0.337081

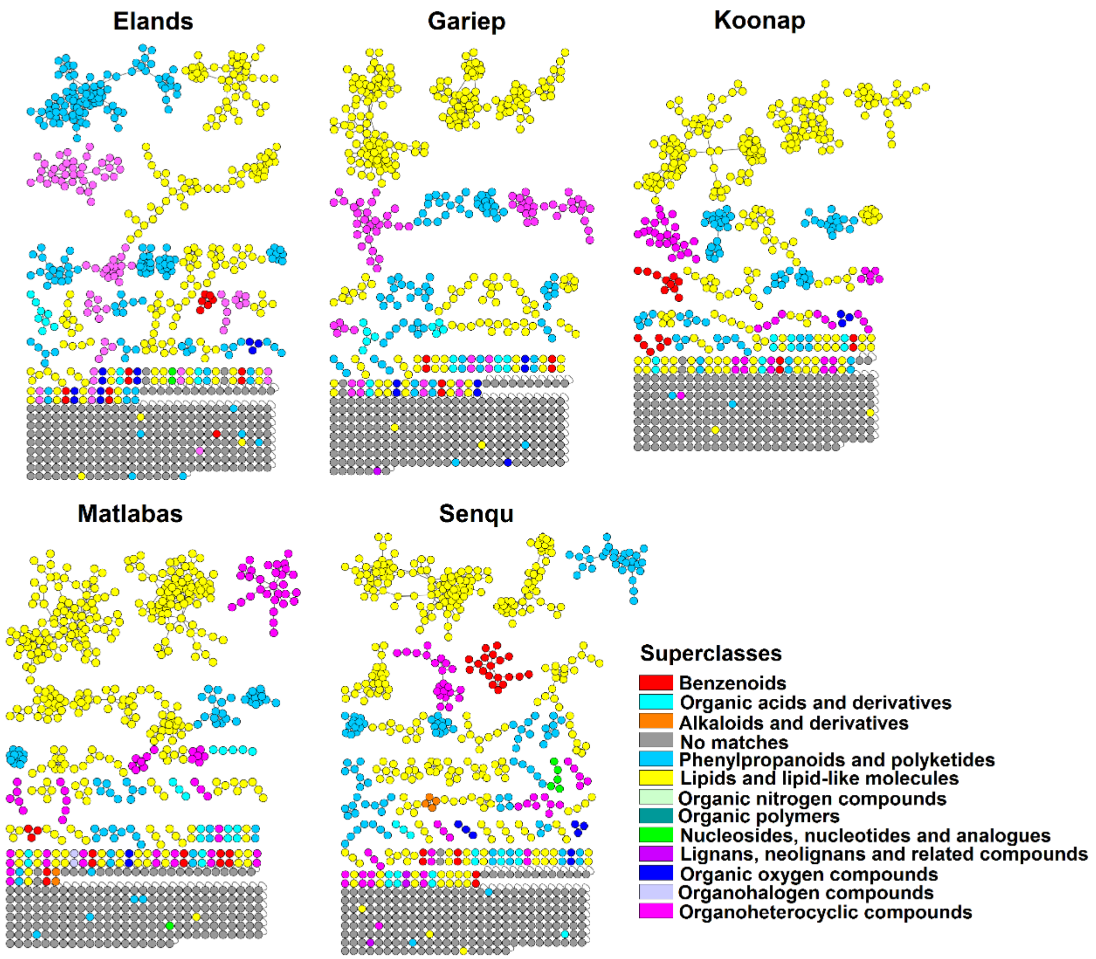

Figure S1: Enhanced molecular networks. The MNs were computed from ESI negative spectra data, display a total of 771, 674, 690, 733 and 776 nodes for the Elands, Gariep, Koonap, Matlabas and Senqu cultivars computed networks, respectively. The coloured nodes are representative of superclasses of putatively annotated metabolites matched to reference spectra in GNPS-housed libraries, while grey nodes represent unknown superclasses (*i.e.,* those with no library matches).

Table S8: Metabolite classes, abbreviations and full names of all targeted and putatively annotated metabolites shown in the sunburst plot (**Figure 3**)

Class	Abbreviations	Full compound name	Fold change
**Amino acids**	Ser	Serine	1
	Ala	Alanine	1
	Asp	Aspartic acid	1
	Thr	Threonine	1
	Pro	Proline	1
	Cys	Cysteine	1
	Val	Valine	1
	OH-Pro	Hydroxyproline	1
	Tyr	Tyrosine	1
	D-Fluo-Phe	D-Fluoro-Phenylalanine	1
	Tyr	Tryptophan	1
**Flavonoids**	Vitexin	Vitexin	1
	Chry-8-glu	Chrysoeriol 8-C-glucoside	1
	Cor	Corymboside	1
	Isoschaftoside	Isoschaftoside	1
	Vicenin-3	Vicenin-2	1
	Rutin	Rutin	1
	Iso-rut	isorhamnetin-3-O-rutinoside	1
	Demethoxy-rut	Demethoxycentaureidin 7-O-rutinoside	1
	Iso-que	Isoquercitirin (same as isoquercetin)	1
	Que-gl	Quercetin-3-(2-glucosylrhamnoside)	1
	Tri-gLu	Tricin-7-glucoside	1
	Violanthin	Violanthin	1
	Tri-flavone	5,6,2'-Trimethoxyflavone	1
	Tricin	Tricin	1
	Api-ara	Apigenin 6-C-I<<-L-arabinopyranosyl-8-C-EC-D-xylopyranoside	1
	Api-glu-ara	Apigenin 6-C-glucoside 8-C-arabinoside	1

	Dihyd-flavone	3,4-Dihydroxyflavone	1
	Scoparin	Scoparin	1
	Apigenin	Apigenin	1
	Luteolin	Luteolin	1
	Apigeninidin	Apigeninidin	1
	Luteolinidin	Luteolinidin	1
	Luteoside	Luteoside	1
	Apigenitrin	Apigenitrin	1
**HCA compounds**	ChlorogenicA	Chlorogenic acid	1
	FerQA	Feruloylquinic acid	1
	3-Fer-suc	3-O-Feruloylsucrose	1
	t-CafA	trans-caffeic acid	1
	FerA	Ferulic acid	1
	Di-CafQA	4,5-Dicaffeoyl quinic acid	1
**Hormones**	ILA	3-Indolelactic acid	1
	I3A	Indole-3-carboxyaldehyde	1
	I3CA	Indole-3-carboxylicacid	1
	IAA	IAA	1
	MEJA	MEJA	1
	SA	SA	1
**Lipids**	Oleo-glyP	1-Oleoylglycerophosphoinositol	1
	HODE	13-HODE	1
	TriHODE i	9,12,13-trihydroxy-10,15-octadecadienoic acid	1
	TriHOME i	5,8,11-trihydroxyoctadec-9-enoic acid	1
	Palm-glyP	1-palmitoylglycerophosphoinositol	1
	DGMG	DGMG 18:2	1
	AzeA	Azelaic acid	1
	KODE	9-KODE	1
	HOTrE	13-HOTrE	1
	DiHOME i	9, 10-DiHOME	1

	DiHOME ii	12, 13-DiHOME	1
	TriHODE ii	(10E,15E)-9,12,13-trihydroxyoctadeca-10,15-dienoic acid (9,12,13-TriHODE)	1
	TriHOME ii	trihydroxyoctadec-9-enoic acid	1
	Hex-gly-pho	1-Hexadecanoyl-sn-glycero-3-phospho-(1'-sn-glycerol)	1
	MGMG	MGMG 18:2 (ADDUCT)	1
**Phenolic alkaloids**	Ave A	Avenanthramide A	1
	Ave B	Avenanthramide B	1
	Hord	Hordenine	1
**Ribonucleoside**	Adenosine	Adenosine	1
	xanthosine	xanthosine	1
**Sugar**	sucrose	sucrose	1
	Melibiose	Melibiose	1
	maltotriose	maltotriose	1
	Raffinose	Raffinose	1
	Stachyose	Stachyose	1
	Acanthoside B	Acanthoside B	1
	Juniperoside III	Juniperoside III	1
	Trehalose	Trehalose	1
	Palatinose	Palatinose	1
**Other**	Pyrenophorol	Pyrenophorol	1
	hyd-hylpthalamide	N-hydroxyethylpthalamide	1
	Endocrocin	Endocrocin	1
	Scutellarioside II	Scutellarioside II	1
	Di-Fer gly	Diferuloyl glycerol	1
	Aco acetate	2,4-dihydroxyheptadec-16-yn-1-yl acetate (Avocadyne acetate)	1
	DiCou-Spermidine	Dicoumaroyl Spermidine	1
	Fraxin	Fraxin	1
	CitricA	Citric acid	1
	Canrenone	Canrenone	1

	benzA der	3-(Benzoyloxy)-2-hydroxypropyl beta-D-glucopyranosiduronic acid	1
	aurapten	aurapten	1
	Pinolidoxin	Pinolidoxin	1
	Arill	Arillatose B	1
	Guanosine	Guanosine	1
	UDP-ace-glu	UDP-N-acetylglucosamine	1
	Gardnerine	Gardnerine	1
	DIMBOA	DIMBOA	1
